# Quantitative detection of the *Ralstonia solanacearum* species complex in soil by qPCR combined with a recombinant internal control strain

**DOI:** 10.1128/spectrum.00210-23

**Published:** 2023-11-15

**Authors:** Wei Chen, Jun-Wei Zhang, Bi-Xia Qin, Hui-Ting Xie, Zhi Zhang, Xiu-Ze Qiao, Shan-Kui Li, Muhammad Asif, Song Guo, Li-Xian Cui, Pei-Pei Wang, Li-Hong Dong, Qing-Gang Guo, Wen-Jun Jiang, Ping Ma, Zhen-Yuan Xia, Can-Hua Lu, Li-Qun Zhang

**Affiliations:** 1 Ministry of Agriculture and Rural Affairs Key Laboratory of Pest Monitoring and Green Management, College of Plant Protection, China Agricultural University, Haidian District, Beijing, China; 2 Plant Protection Research Institute, Guangxi Academy of Agricultural Sciences, Guangxi Key Laboratory of Biology for Crop Diseases and Insect Pests, Xixiangtang District, Nanning, China; 3 Institute of Plant Protection, Hebei Academy of Agriculture and Forestry Sciences, Key Laboratory of IPM on Crops in Northern Region of North China, Ministry of Agriculture and Rural Affairs, IPM Innovation Centre of Hebei Province, Lianchi District, Baoding, China; 4 Yunnan Academy of Tobacco Agricultural Sciences, Wuhua District, Kunming, China; University of Minnesota Twin Cities, Saint Paul, Minnesota, USA

**Keywords:** *Ralstonia solanacearum *species complex, quantitative PCR, large-scale sequence analysis, internal sample process control

## Abstract

The detection of soil-borne pathogens by quantitative PCR (qPCR) has been challenging due to the pronounced influence of soil type on DNA extraction and PCR reactions. In the present study, we developed a novel qPCR system and an internal sample process control (ISPC) strain, RsPC, for the detection of *Ralstonia solanacearum* species complex (RSSC), the pathogens causing bacterial wilt. Specific primers and TaqMan probe were designed based on analyses of 16S rRNA gene sequences from 581 *Ralstonia* genomes, and the RsPC was constructed by insertion of an artificial fragment, which consisted of two fragments from the kanamycin-resistant gene and the *gfp* gene, into the chromosome of a phylogenetically closely related strain, *Ralstonia pickettii* JCM 5969. The qPCR target sequences of RSSC and RsPC shared primers; however, different TaqMan probes were used to distinguish them from each other. The interaction assay between ISPC and target DNA showed no influence on sensitivity when their difference in concentration was <10^4^-fold. We tested 10 different soils with co-spiked RsPC and *R. pseudosolanacearum* LMG 9673 and found comparable recovery efficiencies (REs) of two strains in most samples, and the REs of LMG 9673 after correction by RsPC were closer to theoretical values. The RE of LMG 9673 improved most (2.98-fold) in a heavy clay soil. Similar results were obtained in three representative soils co-spiked with RsPC and LMG 9673 (at three concentrations), *R. solanacearum* NCPPB 325, and *R. syzygii* LLRS-1, respectively . The qPCR system and ISPC strain developed in this study could be applied for the accurate detection of RSSC in soil, and similar ISPCs can be developed in the future for other soil-borne animal and plant pathogens.

**IMPORTANCE:**

DNA-based detection and quantification of soil-borne pathogens, such as the *Ralstonia solanacearum* species complex (RSSC), plays a vital role in risk assessment, but meanwhile, precise quantification is difficult due to the poor purity and yield of the soil DNA retrieved. The internal sample process control (ISPC) strain RsPC we developed solved this problem and significantly improved the accuracy of quantification of RSSC in different soils. ISPC-based quantitative PCR detection is a method especially suitable for the quantitative detection of microbes in complex matrices (such as soil and sludge) containing various PCR inhibitors and for those not easy to lyse (like Gram-positive bacteria, fungi, and thick-wall cells like resting spores). In addition, the use of ISPC strains removes additional workload on the preparation of high-quality template DNA and facilitates the development of high-throughput quantitative detection techniques for soil microbes.

## INTRODUCTION

Bacterial wilt is a destructive disease in Solanaceae crops caused by the *Ralstonia solanacearum* species complex (RSSC), including *R. solanacearum*, *R. pseudosolanacearum*, and *R. syzygii* (1). Other species in the genus *Ralstonia*, including *R. pickettii*, *R. insidiosa*, and *R. mannitolilytica*, are non-pathogenic to plants. The bacterial cells of RSSC can survive in soil, infect host roots through wounds or natural openings, spread systemically in xylem vessels, block water or mineral transportation, and cause plant wilting (2). After harvesting, the bacteria in disease residues are left in the soil and survive for several years under natural conditions (3). As a soil-borne disease, the occurrence and severity of bacterial wilt are related to the bacterial populations in the soil (4). Therefore, quantitative detection of RSSC in soil before sowing could facilitate the prediction of disease risk and planting decisions among growers.

Several methods of quantifying RSSC from natural materials, including soil and plants, have been developed. RSSC forms characteristic white fluidal colonies with pink centers on tetrazolium chloride medium or modified semi-selective medium South Africa after plating (5, 6). However, plating-based methods are time-consuming (at least 3 days) and incapable of detecting cells in the viable but non-culturable state that is observed in harsh conditions, resulting in the underestimation of populations (7 – 9). Conversely, serological methods, including indirect enzyme-linked immunosorbent assay (ELISA) and double-antibody sandwich ELISA (DAS-ELISA), enable rapid detection, although their sensitivity is relatively low (10^5^ CFU∙g^−1^ in soil for indirect ELISA and 10^3^–10^6^ CFU mL^−1^ for DAS-ELISA) (10, 11). Currently, quantitative polymerase chain reaction (qPCR) is the most widely used RSSC detection method because of its rapidity, high sensitivity, and precision (12 – 25). However, qPCR detection is more challenging for microbes in soil samples than microbes in water and plant tissue samples because of numerous interfering factors in soil, such as metal ions and organic compounds (polysaccharides, humic acids, and phenolic substances), which inhibit PCR reactions and reduce detection accuracy (26 – 28). To address the above challenge, diluted DNA extracts are often used as templates, or a non-target DNA is spiked as the internal amplification control (IAC) in the template DNA (27, 29). However, the DNA losses during sample preparation and DNA extraction and purification remain unknown, and excessive dilution of the templates would make the target DNA concentrations lower than the detection sensitivity, resulting in false negative results (27).

**FIG 1:**
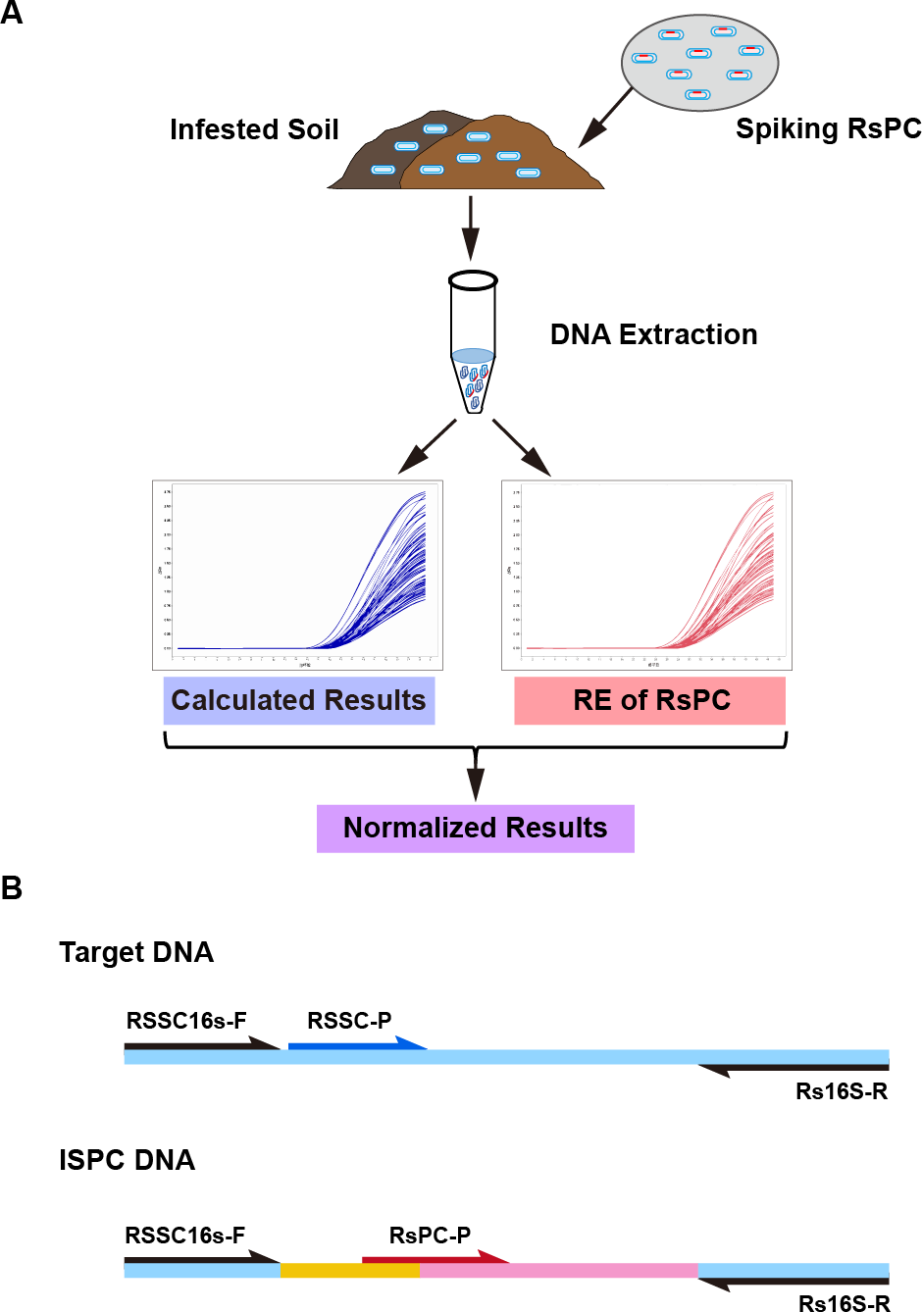
Flowchart of application of RsPC for detection of soil-borne bacteria (**A**) and the structure of target and ISPC DNA (**B**). (A) Fixed amounts of ISPC strain were added to RSSC-infested soil samples before DNA extraction. The efficiency of the entire analytical chain (from sample preparation to qPCR detection) measured using the ISPC strain was used to correct the qPCR detection results of target bacteria. (B) Synthetic ISPC DNA was similar to the target DNA (light blue) and consisted of three parts: specific primer sequences (black), a part of the *gfp* gene (yellow), and a part of the kanamycin resistance gene (pink). The TaqMan probe RsPC-P (red) was designed across *gfp* and kanamycin resistance genes. The TaqMan probe RSSC-P is marked in deep blue.

Internal sample process control (ISPC) is a microorganism spiked in samples before preparation that enables the assessment of the performance of the entire analytical chain (Fig. 1A) (29). The optimal ISPC is an engineered strain containing an artificial DNA fragment as the qPCR target on its genome, which maintains the highest level of similarity with the actual target and specificity in a natural background (29). The early ISPCs were several wild-type viruses used for detecting human viruses in food, water, and clinical samples to avoid false negative results due to PCR inhibition or operation error (30 – 32). Afterward, a series of engineered ISPCs were developed for quantitative detection of pathogenic bacteria (33 – 37). For example, one recombinant ISPC strain was constructed by inserting an artificial sequence into the genome of *Listeria monocytogenes* mutant Δ-*prfA*. Similar recovery efficiencies (REs) were found for a target pathogen (55%) and the ISPC (49%) in artificially contaminated samples, indicating comparable characteristics between the ISPC and the target (34). Another ISPC strain, *Pseudogulbenkiania* sp. NH8B-1D2, containing a kanamycin-resistant gene in one copy of the 23S rRNA genes, was designed for normalizing qPCR results during the detection of water-borne pathogens. The accuracy was significantly improved after normalization (36).

ISPCs have mainly been applied in the detection of bacterial pathogens in clinical, water, and food samples (30 – 40), but have rarely been used for soil samples. Soil is the natural habitat of various plant, human, and animal pathogens and exhibits extremely complex and diverse characteristics, such as soil texture and structure, pH, and biotic activity (41 – 44), many of which considerably influence PCR detection results (28, 45). It remains unclear whether the ISPC improves qPCR detection of soil-borne pathogens significantly. In the present study, we developed an RSSC-specific primer and probe set based on a large-scale genome sequence analysis and constructed a new qPCR system with a genetically engineered ISPC strain for application in RSSC detection.

## RESULTS

### Analysis of *Ralstonia* genomes

In total, 581 *Ralstonia* spp. genome sequences from the National Center for Biotechnology Information (NCBI) genome database were selected for the design of RSSC-specific primers and the TaqMan probe, including 125 complete genome-level, 20 chromosome-level, 96 scaffold-level, and 340 contig-level genome assemblies (Table S1). We re-identified the strains at the species level by Average Nucleotide Identity (ANI) analysis using FastANI. The genome materials covered all six known *Ralstonia* species, including 203 *R*. *pseudosolanacearum* strains, 227 *R*. *solanacearum* strains, 61 *R*. *pickettii* strains, 24 *R*. *syzygii* strains, 12 *R*. *insidiosa* strains, 14 *R*. *mannitolilytica* strains, and 40 unclassified *Ralstonia* spp. strains (Table S1). The unclassified *Ralstonia* spp. strains had less than 95% ANI value to all type strains and were further classified into three subgroups: the Ri/Rpi-like subgroup (33 strains), the RSSC-like subgroup (3 strains), and the “Others” subgroup (4 strains), and their ANI values to all type strains are summarized in Table S2.

The full or partial small ribosomal subunit (16S rRNA) gene of all the genomes was extracted according to annotation. To determine the copy number of the 16S rRNA gene per genome of RSSC species, we analyzed the complete-level genome assemblies. The copy number of the 16S rRNA gene varied among species in the genus *Ralstonia*. Out of the 89 *R. pseudosolanacearum* strains, 88 had 4 copies of 16S rRNA genes per genome, and the remaining strain, YC45, contained 2 copies. Eleven out of 13 *R*. *solanacearum* strains had 3 copies, and the remaining 2 strains (CFBP 8697 and CFBP 8695) had only 1 copy. Most *R. syzygii* strains (14 out of 15) had 3 copies, excluding strain KACC 10722 (2 copies) (Table S1).

### Design of RSSC-specific primers and the TaqMan minor groove binder probe

**FIG 2:**
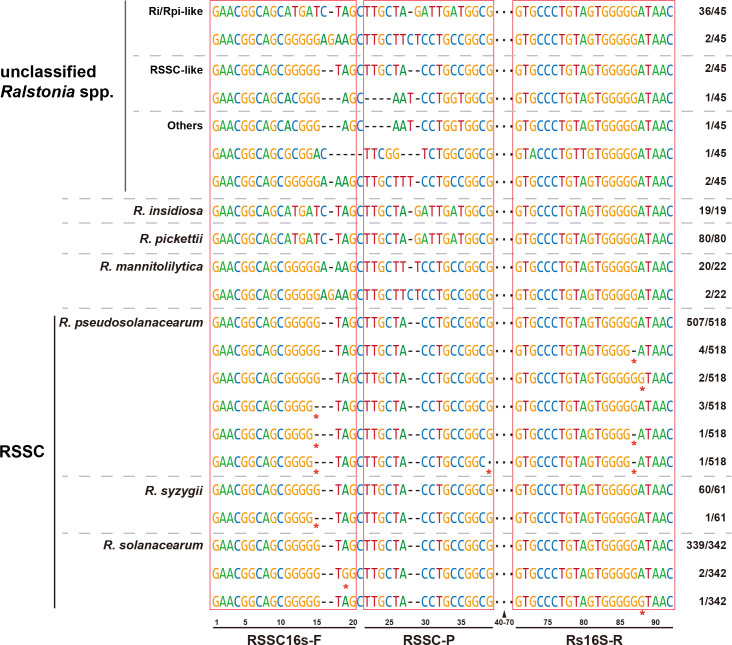
Alignment and analysis of the RSSC-specific region of 16S rRNA gene sequences from 581 *Ralstonia* genomes. The RSSC sequences containing nucleotide variations (labeled with a red star) are listed, and the numbers after each sequence indicate proportions of the sequence in the group. Numbers above the black line indicate the sequence position from 5′-terminal to 3′-terminal, and the black triangle marks the omitted part of the qPCR target sequence. The primers and probe regions are indicated by a red box and labeled at the bottom.

The primer RSSC16s-F and TaqMan probe RSSC-P targeting 16S rRNA gene sequence were designed specifically for RSSC strains, according to alignment results (Fig. 2). Another primer, Rs16S-R, matched to all *Ralstonia* species, was published previously (Fig. 2) (19). Overall, 1,087 16S rRNA gene sequences were extracted from 581 tested *Ralstonia* genomes. The primers RSSC16s-F/Rs16S-R and TaqMan probe RSSC-P matched perfectly with 99.12% of *R. solanacearum* sequences (339/342), 98.36% of *R. syzygii* sequences (60/61), and 97.88% of *R. pseudosolanacearum* sequences (507/518). Only 15 out of 921 RSSC sequences contained nucleotide variations in the primer and probe binding regions (Fig. 2). For *R. pseudosolanacearum*, a single deletion of the 6th cytosine occurred in the Rs16S-R binding regions of four sequences from four strains (T78, T110, FJAT91-F8, and YQ); the 5th thymine of the Rs16S-R binding region was replaced by cytosine in two 16S rRNA gene copies of strain RUN2474; the 15th guanine in the RSSC16s-F binding region was missing in one 16S rRNA gene copy of T25 and two 16S rRNA gene copies of FJAT454.F50-1; and the deletion together with another single deletion of the 6th cytosine in Rs16S-R occurred in another 16S rRNA gene copy of strain FJAT454.F50-1. In strain YC45, deletions of the 6th cytosine in Rs16S-R, the 15th guanine in RSSC16s-F, and the 16th guanine in RSSC-P occurred in the same 16S rRNA gene copy. For *R. solanacearum*, the 17th adenosine of RSSC16s-F was replaced by guanine in two 16S rRNA gene copies belonging to UW256 and PD 3270, and the 5th thymine of Rs16S-R was replaced by cytosine in one 16S rRNA gene copy of UW88. The only nucleotide variation in *R. syzygii* was the deletion of the 15th guanine in the RSSC16s-F binding site in one 16S rRNA gene copy of SL3022. The nucleotide variations could be the product of natural evolution or may be caused by polymerase errors during PCR or sequencing errors because they were abundant in homopolymer regions of genomes sequenced by PacBio and Nanopore technologies (46, 47). Such mismatches are not expected to significantly influence our specific qPCR because they are few in number and not located at the 3′ ends of the primers.

In contrast, more mismatches close to the 3′ end were found at the position of the primer RSSC16s-F in non-RSSC strains. Primer RSSC16s-F exhibited six mismatches to sequences of *R. pickettii* or *R. insidiosa* between the 10th and 16th nucleotides and 2–3 mismatches to *R. mannitolilytica* between the 15th and 17th nucleotides (Fig. 2). Moreover, the TaqMan probe RSSC-P had five mismatches with *R. pickettii* or *R. insidiosa* and two to three mismatches with *R. mannitolilytica* (Fig. 2).

In the “unclassified *Ralstonia* spp.” group, two out of three 16S rRNA gene sequences of the “RSSC-like” subgroup had no mismatches to primers or probe sets (Fig. 2). The remaining 16S rRNA gene copy had three mismatches with primer RSSC16s-F and eight mismatches with probe RSSC-P. In the “Ri/Rpi-like” subgroup, 36 sequences extracted from 31 strains had similar mismatches with the primer and probe sets as *R. pickettii* or *R. insidiosa*. Two other sequences from two strains had three mismatches with primer RSSC16s-F and three mismatches with probe RSSC-P. In the “Others” subgroup, two sequences had two mismatches with primer RSSC16s-F and two mismatches with probe RSSC-P. One sequence had three mismatches with primer RSSC16s-F and eight mismatches with probe RSSC-P, and another sequence had five mismatches with primer RSSC16s-F, six mismatches with probe RSSC-P, and one mismatch with primer Rs16S-R (Fig. 2).

The specificity of the primer and probe set was checked *in silico* by BLAST, and the primer and probe set did not match strains of other genera. The specificity was experimentally tested using the genome DNA of the type strains of *R. insidiosa*, *R. pickettii*, *R. mannitolilytica*, and *R. pseudosolanacearum*. No signal was detected when the genome DNA of *R. insidiosa* LMG 21421 and *R. pickettii* JCM 5969 served as templates. The detection limit of *R. mannitolilytica* LMG 6866 was 50 pg of genome DNA per reaction (about 10^4^ genomes) (Table S3). For *R. pseudosolanacearum* LMG 9673, the sensitivity was 50 fg genome DNA per reaction (about eight genomes), about 10^3^-fold higher than for *R. mannitolilytica* LMG 6866 (Table S3).

### Construction of the *Ralstonia solanacearum* species complex ISPC strain RsPC

The ISPC strain for RSSC detection was constructed by mini-Tn7-mediated integration of a single-copy ISPC DNA fragment into the chromosome of *R. pickettii* JCM 5969. The resulting ISPC strain, RsPC, was confirmed by PCR amplification and sequencing (data not shown). The TaqMan minor groove binder (MGB) probe RsPC-P was designed based on the junction region between *gfp* and kanamycin-resistant genes and is specific to RsPC (Fig. 1B; Table 1). BLAST analysis revealed that no other nucleotide sequences matched with RsPC-P, and the qPCR detection using RsPC-P as the probe resulted in a high fluorescent signal only when the gDNA of RsPC, but not its wild-type strain, *R. pickettii* JCM 5969, and target bacterium *R. pseudosolanacearum* LMG 9673, served as templates (data not shown). Therefore, the target DNA and its corresponding ISPC DNA in our qPCR reaction could be distinguished using different TaqMan probes after amplification by the same primer set, RSSC16s-F/Rs16S-R. In addition, both PCR reactions produced amplicons with the same length (88 bp) to maintain similar PCR efficiencies.

**TABLE 1:** Sequences of primers, probes, and ISPC DNA used in the present study

Oligonucleotide name *^a^*	Sequence (5′–3′)	Length (nt)	Resource
RSSC16s-F	GAACGGCAGCGGGGGTAG	18	This study
Rs16S-R	GTTATCCCCCACTACAGGGCAC	22	(19)
RSSC-P	FAM-TTGCTACCTGCCGGCG-NFQ-MGB*^b^*	16	This study
RsPC-P	FAM-ACCTGTCTGACCGCTTC-NFQ-MGB*^b^*	17	This study
RSSC16s-F-EcoRI	AT**GAATTC**GAACGGCAGCGGGGGTAG *^c^*	26	This study
Rs16s-R-EcoRI	AT**GAATTC**GTTATCCCCCACTACAGGGCAC *^c^*	30	This study
Tn7 check glmS-F	ATCCAGGTGATCCGCATGC	19	This study
Tn7 check R	TGGGAACTGGGTGTAGCGTCGTAA	24	This study
ISPC DNA	**GAACGGCAGCGGGGGTAG** *ACAACCATT* ***ACCTGTC*** **TGACCGCTTC** CTCGTGCTTTACGGTATCGCCG **GTGCCCTGTAGTGGGGGATAAC** ^*d*^	88	This study

^*a*^ F, forward primer; R, reverse primer; P, TaqMan probe.

^*b*^ The TaqMan probes were labeled with FAM fluorophore at the 5′ end and the non-fluorescent quencher (NFQ)-MGB group at the 3′ end.

^*c*^ The bold letters represent restriction sites.

^*d*^ The bold letters represent the primer and probe sequences. The italic letters represent the sequence from the *gfp* gene, and the underlined letters represent the sequence from the kanamycin resistance gene.

### Standard curves of target and ISPC DNA

**FIG 3:**
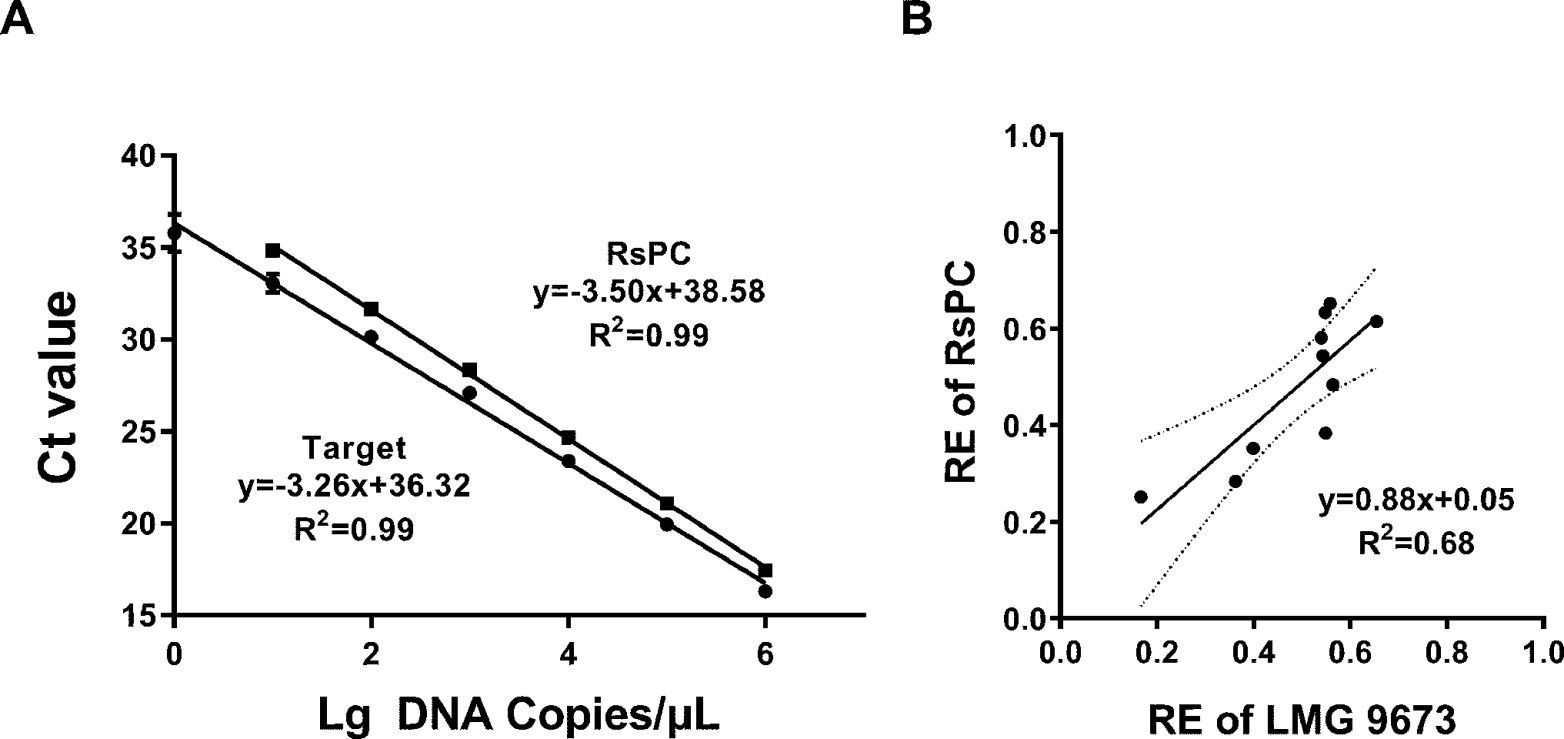
(**A**) Standard curves of cycle threshold value (Ct) plotted against the log value of the DNA concentration of gradient-diluted recombinant plasmids. (**B**) Quantitative relationships between REs of LMG 9673 and RsPC in 10 different samples. In A, squares represent the data for ISPC DNA, and circles represent the target DNA. The concentration of plasmids ranged from 10^0^ to 10^6^ copies/μL, and 5 µL DNA solutions were used as a template. In B, dotted lines indicate 95% confidence bands. Regression analysis was conducted using GraphPad Prism 9 (*P* < 0.05). Slope, intercept, and *R* ^2^ are shown in the equations.

Serial dilutions of the linearized recombinant plasmids pMD18-Rs16S and pMD18-RsPC were used as qPCR templates to construct the standard curves (Fig. 3A). The qPCR successfully detected target sequences of 5 × 10^0^–5 × 10^6^ copies and ISPC sequences of 5 × 10^1^–5 × 10^6^ copies per reaction, with a good *R* ^2^ value of 0.99 for both target and ISPC sequences. The PCR amplification efficiencies of target (102.6%) and ISPC sequences (93.1%) were both within the optimal range (90%–110%) (48).

### Interaction between target DNA and ISPC DNA

The RSSC strains and RsPC shared primer sets and reaction conditions to minimize differences during the qPCR amplification. However, two DNA templates in the same reaction mixture may compete for primers, deoxy-ribonucleoside triphosphate (dNTPs), and polymerase and consequently reduce detection efficiency and sensitivity, especially when the concentrations of two templates differ greatly. To avoid the above, we detected the interaction effects between the two templates and determined the appropriate RsPC addition amount. We mixed the target and ISPC plasmids at different ratios and detected the qPCR sensitivity of one of them against different backgrounds (Table 2). The results indicated that the qPCR sensitivities were not significantly affected when the ratios of two DNA fragments were less than 10^4^-fold. For target DNA, the detection sensitivity was 5 × 10^0^ copies per reaction in the absence of ISPC DNA, which did not change in the presence of low amounts of ISPC DNA (5 × 10^0^ to 5 × 10^4^ copies). When the background ISPC DNA increased to 5 × 10^5^ and 5 × 10^6^ copies, the qPCR sensitivities of target DNA decreased from 5 × 10^0^ to 5 × 10^1^ and 5 × 10^2^ copies per reaction, respectively (Table 2). Similar trends were observed when ISPC DNA was detected against the target DNA background (Table 2). Therefore, the difference in qPCR target fragment abundance between artificially added ISPC and target bacteria in the tested soil samples should not exceed 10^4^-fold.

**TABLE 2:** Assessment of the qPCR sensitivity of the target or ISPC DNA against the other fragment as background

Target DNA as background (copies/reaction) *^a^*	ISPC sensitivity (copies/reaction)^*a*^	ISPC DNA as background (copies/reaction)^*b*^	Target sensitivity (copies/reaction)^*b*^
5 × 10^7^	5 × 10^3^	5 × 10^6^	5 × 10^2^
5 × 10^6^	5 × 10^2^	5 × 10^5^	5 × 10^1^
5 × 10^5^	5 × 10^1^	5 × 10^4^	5 × 10^0^
5 × 10^4^	5 × 10^1^	5 × 10^3^	5 × 10^0^
5 × 10^3^	5 × 10^1^	5 × 10^2^	5 × 10^0^
5 × 10^2^	5 × 10^1^	5 × 10^1^	5 × 10^0^
5 × 10^1^	5 × 10^1^	5 × 10^0^	5 × 10^0^
0	5 × 10^1^	0	5 × 10^0^

^*a*^ The target DNA (0 to 5 × 10^7^ copies) was used as background, and the ISPC DNA was detected.

^*b*^ The ISPC DNA (0 to 5 × 10^6^ copies) was used as background, and the target DNA was detected.

### Recovery efficiencies of *R. pseudosolanacearum* LMG 9673 and RsPC in 10 different soil samples

RsPC was applied to evaluate and normalize REs of *R. pseudosolanacearum* LMG 9673 in 10 soil samples with different texture, pH, and organic matter (OM) content (Table 3). No signal of target or ISPC DNA could be detected from all the soil samples by qPCR before artificial inoculation (data not shown). LMG 9673 and the ISPC strain were co-spiked in each soil with final amounts of 6.80 × 10^4^ and 1.70 × 10^5^ CFU/g, respectively, and parallel qPCR was performed for both strains. The copy number of the 16S rRNA gene was four copies per genome in LMG 9673 and was used for calculating the genome amount of LMG 9673.

**TABLE 3:** Evaluation of recovery efficiencies of LMG 9673 and RsPC in 10 soil samples^*g*^

Locations	Soil type^*a*^	pH	OM^*b*^ (%)	Clay (%)	Silt (%)	Sand (%)	LMG 9673					RsPC			Corrected RE of LMG 9673 (%)	Relative accuracy before normalization	Relative accuracy after normalization
							RE (%)^*c*^		Std (%)^*d*^			RE (%)		Std (%)			
Wulanchabu	Sandy loam	8.17	1.19	2.760	12.460	84.780	55.81	a^*e*^	7.38		65.15		a	9.29	85.67	0.84	0.22
Shanghai	Sandy loam	5.37	2.98	2.846	32.947	64.207	65.44	a	14.39		61.44		abc	14.74	106.52	0.61	-0.09
Xingtai	Sandy loam	7.37	2.24	3.328	36.587	60.085	54.01	ab	10.29		58.04		abc	41.53	93.07	0.89	0.10
Jiayuguan^*f*^	Sandy loam	8.08	1.85	4.268	40.653	55.079	54.92	a	2.24		38.30		bcde	3.06	143.40	0.86	-0.52
Bayannaoer	Loam	7.58	2.16	4.971	42.417	52.612	39.88	bc	3.58		35.25		cde	5.00	113.12	1.33	-0.18
Haerbin	Silty loam	6.83	3.56	2.065	54.846	43.089	36.21	c	11.35		28.37		de	6.46	127.62	1.47	-0.35
Shenyang	Silty loam	6.26	2.45	2.082	49.850	48.068	54.32	ab	14.02		54.32		abcd	14.54	100.00	0.88	0.00
Chengdu	Silty loam	5.33	4.10	3.255	65.707	31.038	54.85	a	2.90		63.30		ab	6.71	86.66	0.87	0.21
Nanchang	Silty loam	5.76	5.35	5.937	54.713	39.350	56.41	a	4.81		48.34		abcde	8.40	116.68	0.83	-0.22
Kunming	Heavy clay	5.46	3.13	65.16	23.990	10.849	16.69	d	4.90		25.13		e	4.94	66.40	2.58	0.59

^*a*^ Soil textures were determined according to the international system of soil texture classification.

^*b*^ Organic matter.

^*c*^ Recovery efficiency (RE).

^*d*^ Standard deviation.

^*e*^ Lowercase letters after REs indicate significant differences between REs of the same strain in 10 different soil samples by one-way analysis of variance (*P* < 0.05).

^*f*^ A significant difference between REs of LMG 9673 and RsPC was only found in the Jiayuguan soil sample using the *t*-test (*P* < 0.05).

^*g*^ DNA extraction was performed using DNeasy PowerSoil Kit. REs and standard deviations were calculated based on three biological replicates. QPCR detections for each biological replicate were performed in three technical replicates.

Significant differences in REs were observed for the same strain among different soil samples (Table 3), highlighting the significant influence of soil properties on qPCR detection results. The target strain was recovered from soil samples with an efficiency of 16.69 ± 4.90% to 65.44 ± 14.39%. The highest efficiency (65.44%, Shanghai, sandy loam) was approximately 3.92-fold of the lowest (16.69%, Kunming, heavy clay). REs of the ISPC strain were less variable, ranging from 25.13 ± 4.94% to 65.15 ± 9.29%; however, the general trend was similar to that of the target strain. Both strains had the lowest REs in heavy clay soil (Kunming, 16.69% for the target strain and 25.13% for ISPC) and the highest REs in sandy loam soil (65.44% for target strain in the Shanghai soil sample and 65.15% for ISPC in the Wulanchabu soil sample). No significant differences in REs were observed between the target and ISPC strains in the same soil sample, excluding the one from Jiayuguan (Table 3). According to the regression analysis results, there was a strong positive correlation (*R* ^2^ = 0.68) between the REs of LMG 9673 and RsPC (Fig. 3B). The slope value of 0.88 was tested by an extra-sum-of-squares F-test and was not significantly different from 1.00, indicating that the RE values of LMG 9673 were statistically equivalent to those of RsPC (Fig. 3B). The comparable performance of the two strains in qPCR detection demonstrated the feasibility of the ISPC strain in the correction of qPCR quantification.

The ISPC was employed to adjust the RE of target bacterium LMG 9673 in the same soil sample and improved detection accuracy significantly (Table 3). Compared with the non-corrected REs, all the corrected REs of LMG 9673 were closer to the theoretical value (100%). Notably, the RE of LMG 9673 from the heavy clay soil (Kunming) increased approximately three-fold, from 16.69% to 66.40%, after normalization. To illustrate the results more intuitively, relative accuracy [log_2_(1/RE)] was employed to indicate the deviation of the test results from their theoretical values. The higher degree of the relative accuracy value is closer to 0, where the RE value is 100% (Table 3). Compared to the uncorrected data, the relative accuracy of all samples was much closer to 0 after normalization, indicating a marked improvement in detection accuracy. The results demonstrate the important role of the ISPC strain in improving qPCR detection accuracy, especially in samples with low REs (Table 3).

We conducted regression analysis to examine the impact of physicochemical characteristics on recovery efficiencies (Fig. S1). A significantly negative correlation was found between the clay particles and the REs of LMG 9673, while a significantly positive correlation was found between the sand particles and the REs of LMG 9673. The effect of the clay or sand particles on the REs of RsPC was not statistically significant but exhibited a moderate correlation. Other characteristics, including pH, content of organic matter, and silt particles, showed no or weak correlation to the REs of LMG 9673 and RsPC.

### Validation of the ISPC-based qPCR detection of RSSC at different concentrations in three representative soil samples

The REs were measured in three representative soil samples (Kunming, Chengdu, and Wulanchabu) containing *R. solanacearum* NCPPB 325, *R. syzygii* LLRS-1, or *R. pseudosolanacearum* LMG 9673 at different concentrations together with RsPC. Due to the incomplete genome of NCPPB 325, it is not possible to confirm the copy number of the 16S rRNA gene. We assumed three copies per genome to calculate the genome number of NCPPB 325, as this was observed in 84.62% (11/13) of *R. solanacearum* strains. The copy number of the 16S rRNA gene was three in LRRS-1, according to genome annotation.

Consistent with the results above, the REs of all strains in Kunming soil were lower than those observed in the other two soils across most combinations (Table 4; Table S4). The REs showed no significant difference between target strain and RsPC in all combinations, except for 6.20 × 10^3^ CFU/g of LMG 9673 combined with RsPC in Wulanchabu soil. After normalization, the REs of the target strain exhibited a significant increase, especially for LLRS-1 in heavy clay soil (Table 4; Table S4). This clearly demonstrated the feasibility of RsPC for accurately quantifying RSSC at varying concentrations.

**TABLE 4:** Evaluation of REs of LMG 9673 and RsPC in Kunming, Chengdu, and Wulanchabu soil samples^*e*^

Locations	Soil type^*a*^	Population of target (CFU)	LMG 9673			RsPC		Corrected RE of LMG 9673 (%)	Relative accuracy before normalization	Relative accuracy after normalization
			RE (%)^*b*^	Std (%)^*c*^		RE (%)	Std (%)			
Kunming	Heavy clay	1.55 × 10^5^	21.42	7.81		27.06	7.45	79.15	2.22	0.34
		1.55 × 10^4^	27.72	7.50		32.09	3.93	86.37	1.85	0.21
		1.55 × 10^3^	52.40	39.61		29.44	5.42	177.99	0.93	-0.83
Chengdu	Silty loam	1.55 × 10^5^	64.40	2.27		72.83	7.92	88.42	0.63	0.18
		1.55 × 10^4^	61.18	18.18		58.68	19.39	104.27	0.71	-0.06
		1.55 × 10^3^	61.43	11.50		61.07	8.14	100.60	0.70	-0.01
Wulanchabu	Sandy loam	1.55 × 10^5^	58.10	7.47		59.14	9.80	98.24	0.78	0.03
		1.55 × 10^4^	58.75	10.35		76.78	19.16	76.51	0.77	0.39
		1.55 × 10^3^	45.69^*d*^	4.48		70.36	9.80	64.94	1.13	0.62

^*a*^ Soil textures were determined according to the international system of soil texture classification.

^*b*^ Recovery efficiency.

^*c*^ Standard deviation.

^*d*^ The significant difference between REs of LMG 9673 and RsPC in each combination was only found when LMG 9673 (1.55 × 10^3^ CFU) and RsPC (1.14 × 10^4^ CFU) were co-spiked in Wulanchabu soil using the *t*-test (*P* < 0.05).

^*e*^ Target strain LMG 9673 was inoculated at three concentrations (1.55 × 10^5^, 1.55 × 10^4^, and 1.55 × 10^3^ CFU) together with RsPC (1.14 × 10^4^ CFU) with three biological replicates. DNA extraction was performed using the DNeasy PowerSoil Kit. REs and standard deviations were calculated based on three biological replicates. QQPCR detections for each biological replicate were performed in three technical replicates.

## DISCUSSION

In the present study, we developed a new qPCR system for quantitative detection of RSSC in soil, which is characterized by an RSSC-specific primer/probe set designed based on 1,087 16S rRNA gene sequences extracted from 581 *Ralstonia* genomes and an ISPC strain constructed for monitoring errors during sample processing and qPCR reactions. Several qPCR methods for targeting RSSC or its individual pathogenic species, biovars, or phylotypes have been reported. In addition, primers and probes for RSSC strains have been designed based on the 16S rRNA gene, endoglucanase gene, and upstream region of the UDP-3-*O*-acyl-GlcNAc deacetylase gene (12, 15, 16, 19), and primers for distinguishing races, biovars, and phylotypes have selected more specific amplification targets, such as phage tail S superfamily sequence, specific AFLP fragment, and ITS region (12 – 14, 17 – 19). Typically, most *Ralstonia* qPCR primers and probes have been designed based on sequence analysis of a limited number of *Ralstonia* strains. However, as the number of sequenced genomes increases, some published primers might be found to be less specific. A recent *in silico* analysis based on 192 RSSC genomes indicated that false positive or false negative results were possible in previous PCR assays because of a lack of sufficient sequencing information to guarantee primer specificity and coverage (49). Similarly, a study of *Pseudomonas aeruginosa* revealed that a previously developed method based on restricted numbers of sequences could only detect 82.1% of tested strains, and the novel assay designed using 1,000 genomes of *P. aeruginosa* and 1,017 genomes of other pathogens could detect all tested strains (50). Therefore, it was necessary to conduct large-scale DNA sequence analysis to guarantee the coverage and specificity of primers and probes.

However, some of the bacterial genomes downloaded from public databases could have been incorrectly classified (51). Therefore, we re-identified *Ralstonia* genomes in the present study based on the ANI value before sequence alignment and primer design to avoid potential mistakes. We found that 40 out of 581 genomes had ANI values <95% for all type strains, among which five strains in the Others subgroup are likely to be new species because their ANI values were all <90% for all type strains. Despite the lack of pathogenicity data, the strains appeared to be non-pathogenic as the genes necessary for pathogenicity, such as the T3SS system, were not found in their genomes (data not shown). Strains belonging to the Others and Ri/Rpi-like subgroups should not affect our specific RSSC detection because of the significant mismatches in their primer and probe sequences. For the RSSC-like subgroup, two strains (FJAT-462 and FJAT-452) had the highest ANI values to LMG 9673 (94.51% and 94.67%, respectively), and RSSC-specific region sequences were the same as those of LMG 9673. We speculated that the two strains belonged to *R. pseudosolanacearum*; however, the speculation needs to be supported by more data from other tests. Sequence analysis results indicated that the remaining strain ACH732_UW629 of the RSSC-like subgroup could not be detected by our primer and probe set, although it had the highest ANI value (93.50%) of the type strain of *R. syzygii*. The strain potentially belonged to a new species other than *R. syzygii*. In addition, it was likely plant pathogenic because it was isolated from tomato and had genes encoding pathogenic factors; for example, the T3SS system and the extracellular polysaccharide regulator gene, *epsR*, were found in its genome.

In most cases, bacterial wilt in a given plot was caused by a single RSSC species; however, a specific field or crop might be infected by more than one species (52 – 54). Compared with species-specific detection, our approach could avoid underestimation of the pathogen population in a field infected by multiple species. The 16S rRNA gene was selected as the qPCR target not only because of the apparent sequence difference between the RSSC and non-RSSC strains but also because of its multi-copy nature in the genus *Ralstonia*. The advantage of the multi-copy gene as a qPCR target is that it improves detection sensitivity; nevertheless, attention should be paid to the differences in 16S rRNA gene copy numbers among RSSC species. When converting qPCR results (copies/g) to bacterial density (genomes/g), the 16S rRNA gene copy number of bacterial species in the tested sample should be considered (Table S1).

Although the primer and probe were highly specific for RSSC, we still found weak positive responses when detecting the non-target strain *R. mannitolilytica* LMG 6866. The qPCR sensitivity of LMG 6866 was 50 pg of genome DNA per reaction, equivalent to 7.56 × 10^5^ CFU per gram of soil in our system, without considering DNA recovery efficiency, which is much lower than that of *R. pseudosolanacearum* LMG 9673 (equivalent to 6.24 × 10^2^ CFU per gram of soil). The false-positive results could only occur when the population of *R. mannitolilytica* was more than 7.56 × 10^5^ CFU per gram of soil and at least about 1,200-fold higher than that of *R. pseudosolanacearum*. We speculated that it would not interfere with the detection of RSSC in most cases because the detection limit of *R. mannitolilytica* was much higher.

qPCR detection of microbes in soil has been considered problematic when compared with other frequently studied microbial habitats, such as water, air, food, plant, and animal tissues, because DNA extraction quality varies with soil type and many PCR-inhibiting compounds are co-extracted with DNA (such as humic acids, polysaccharides, phenolic compounds, and heavy metals) (27, 28, 55). A potential solution is inoculating the target pathogen into soil samples at a serial concentration and constructing a standard curve of Ct values against inoculated populations (56). Such a standard curve effectively minimizes the influence of soil DNA extraction and PCR inhibitors; however, it is labor-intensive when applied for high-throughput analysis because such standard curves need to be established for each soil sample separately. We addressed the problem using ISPC technologies and developed the first ISPC strain for quantitative detection of the soil-borne phytopathogenic bacteria RSSC. The resulting ISPC strain had a high degree of similarity to the actual target because it was constructed by integrating the artificial ISPC DNA into the chromosome of *R. pickettii* JCM 5969, a species closely related to RSSC, and it had PCR primers identical to the target. The high similarity between ISPC and RSSC targets ensured that they were similarly affected during soil pretreatment, DNA extraction, and PCR amplification, resulting in similar detection efficiencies. The reliability of the RsPC was supported by the comparable performance of RsPC to LMG 9673 in 10 different soils and LMG 9673, NCPPB 325, and LLRS-1 at varying concentrations in three representative soils (Table 3 and 4 ; Table S4). The results of the regression analysis revealed statistically identical slopes to 1.00 and confirmed comparable performance of RsPC to the actual target in most of the tested soils (Fig. 3B).

The REs (for both target and ISPC strains) varied greatly among the 10 different soils, probably due to their different soil properties. Some soil types affected qPCR detection considerably. For example, the RE values in the heavy clay soil sample (Kunming) were much lower than those in others (16.69% for LMG 9673 and 25.13% for RsPC) (Table 3). Clay particles and high OM content reportedly influence qPCR detection of soil microorganisms considerably (55, 57, 58). Specifically, clay particles absorb DNA, resulting in low-yield DNA extraction (55), whereas high OM content, such as humic acid, causes strong PCR inhibition (58). The OM contents in our soil samples ranged from 1.19% to 5.35%, which resulted in only slight PCR inhibition (57, 59). Therefore, the high clay particle contents were probably the major cause of low REs in Kunming soil. The regression analysis of the soil physicochemical characteristics with REs of LMG 9673 and RsPC further confirmed that the clay/sand particle content significantly influenced the REs of LMG 9673 and RsPC in this study. Consequently, the ISPC strain RsPC is particularly suitable for resolving the above challenges associated with different soil properties, as it enables monitoring of the impacts of DNA loss and PCR inhibitory factors on qPCR detection and compensates by normalizing the measured target signal.

The concentration of ISPC used in soil samples is a key factor influencing detection. In our qPCR assay, amplification of test DNA was strongly inhibited when the concentration of background DNA was 10^4^-fold higher than that of the tested DNA. Similar results were observed in *L. monocytogenes* detection using a competitive IAC, where IAC amplification (25 copies per reaction) was inhibited when mixed with 1.55 × 10^5^ copies of pathogen target DNA but not 1.55 × 10^3^ or 1.55 × 10^1^ copies of pathogen target (60). The inhibition was probably caused by the insufficient reagents in the PCR mixture, which further led to a decrease in PCR efficiency and detection sensitivity. In addition, the level of competition might vary across different microbial targets and qPCR reaction systems. Therefore, the range of concentrations used for ISPC should be validated before detection. Considering the population of RSSC in the infected field was in the 10^2^–10^7^ genomes/g soil range (16, 61) and the suggested concentration of ISPC strain to avoid the potential competition was in the 10^4^–10^5^ CFU/g soil range. As a soil-borne disease, the risk of bacterial wilt is closely related to the RSSC population in soil before sowing or transplanting; however, the threshold population has not yet been determined (62). The ISPC strain could facilitate the resolution of the problem, especially when the threshold should be determined in multiple locations with different soil types.

Soil is the natural habitat of many important plant and human pathogens (63 – 65). In addition to bacterial pathogens, ISPC is suitable for the detection of fungi, oomycetes, protozoa, nematodes, and other organisms in soil, especially those with thick cell walls that are difficult to crack, such as endospores of Gram-positive bacteria and various resting structures of fungi. This study demonstrates ISPC strain RsPC as a powerful tool that could improve the accuracy of bacterial pathogen quantification in soil. Similar ISPCs developed in the future, especially those genetically modified in eukaryotic cells, could facilitate the accurate detection of a wider variety of soil microorganisms.

## MATERIALS AND METHODS

### Bacterial strains and plasmids

The strains and plasmids used are listed in Table 5. The *Ralstonia* strains and their derivatives were cultured in Bacto-Agar and glucose (BG) medium or casamino acid peptone glucose (CPG) agar at 28°C (6, 66). The *Escherichia coli* DH5α was cultured in Luria-Bertani medium at 37°C. The final concentrations of ampicillin and kanamycin were 50 µg/mL.

**TABLE 5:** Bacterial strains and plasmids used in this study

Strain or plasmid	Relevant characteristics	Source
Strains		
*Escherichia coli*		
DH5α	F-*recA1 endA1 hsdR17 supE44 thi-1 gyrA96 relA1Δ (argF-lacZYA) I169Φ80lacZ ΔM15*	Lab collection
DH5α(λ-pir)	F-*recA1 endA1 hsdR17 supE44 thi-1 gyrA96 relA1Δ (argF-lacZYA) I169Φ80lacZ ΔM15 λ-pir*	Lab collection
*Ralstonia pickettii*		
JCM 5969^T^	Wild-type	(67)
RsPC	JCM 5969-Tn7-ISPC DNA; Km^r^	This study
*R. insidiosa*		
LMG 21421^T^	Wild-type	(68)
*R. mannitolilytica*		
LMG 6866^T^	Wild-type	(69)
*R. pseudosolanacearum*		
LMG 9673^T^	Wild-type	(1)
*R. solanacearum*		
NCPPB 325^T^	Wild-type	(1)
*R. syzygii*		
LLRS-1	Wild-type	(70)
Plasmids		
pMD18-T	ColE1 origin, cloning vector; Ap^r^	Takara
pMD18-Rs16S	pMD18 carrying the RSSC target DNA sequence	This study
pMD18-RsPC	pMD18 carrying the ISPC DNA	This study
pCPP6529	pUC18R6KT-Tn*7*T-Km; Ap^r^, Km^r^	(71)
pCPP6529-ISPC	pCPP6529 carrying the ISPC DNA	This study
pTNS2	mobilizable helper plasmid encoding only the specific TnsABC +D transposition, *ori*R6K, *oriT*; Ap^r^	(72)

### Primer and probe design

All genomes of *Ralstonia* spp. were collected from the NCBI genome database. Those genomes reported to be contaminated, too small, or too large were removed. In the cases of genomes with several versions, the latest one was used. To avoid misclassification, ANI analysis of the strains was performed using FastANI v1.33 (73). The ANI threshold value for species demarcation was set at 95% identity for type strains of *Ralstonia* species (74). The strains with less than 95% identity to all-type strains were grouped as unclassified *Ralstonia* spp. Unannotated genomes were annotated using Prokka v1.12 (75), and 16S rRNA gene sequences of all genomes were extracted according to the annotation and aligned using ClustalW in MEGA 5.2 (76). Genomes that contained no RSSC-specific regions of the 16S rRNA gene were discarded. Finally, 581 genomes were selected to design the primer and probe (Table S1). To analyze the number of rRNA genes, all RSSC genomes were annotated using Prokka v1.12, and the number of rRNAs was analyzed by Barrnap (version 0.6). The RSSC-specific primer RSSC16s-F and TaqMan probe RSSC-P were designed according to alignment results (Table 1). Another primer, Rs16S-R, which matched all species of *Ralstonia*, was published previously (19). The specificity of the primer and probe sets was checked *in silico* using BLAST (77) and experimentally tested using the genome DNA of *R. pseudosolanacearum* LMG 9673 and type strains of non-phytopathogenic *Ralstonia* species.

Primers and probes used in the present study are listed in Table 1. All the primers were synthesized by Azenta (Suzhou, China). TaqMan probes were synthesized by Tsingke (Beijing, China) and labeled with FAM at the 5′-terminal and a MGB group with an NFQ at the 3′-end.

### Soil sample collection and preparation

Ten soil samples were collected from different locations in China. Soil was collected using a soil sampler (2.0 cm in inner diameter) together with plant roots from a depth of 20 cm in agricultural fields from 2011 to 2017. The coordinates of latitude and longitude were recorded for partial soil samples. After sampling, the soils were air-dried and stored at 20°C–25°C before use. Soil physicochemical characteristics were analyzed by Sinochem Yantai Crop Nutrition Company (Yantai, China). The information on soil samples is listed in Table S5.

### DNA extraction

Bacterial genomic DNA was extracted using the TIANamp Bacteria DNA Kit (Tiangen, Beijing, China). Soil DNA extraction was carried out using the DNeasy PowerSoil Kit (Qiagen, Germany) or DNeasy PowerSoil Pro Kit (Qiagen, Germany) according to the manufacturer’s instructions. For RE evaluation experiments, RsPC and *Ralstonia* strains were cultured in liquid BG medium at 28°C for 24 hours, washed using sterilized distilled water, and spiked into the soil before DNA extraction.

### Construction of ISPC strain RsPC

The qPCR target fragment of RSSC was modified and used to construct the ISPC strain. The sequence between specific primers was replaced by an artificial substitute sequence and consisted of two fragments from the kanamycin-resistant gene and the *gfp* gene (Fig. 1B; Table 1). This ISPC-specific fragment was synthesized by Tsingke (Beijing).

The ISPC strain RsPC was constructed using the mini-Tn7 system (71). The synthesized ISPC DNA was amplified using primers RSSC16s-F-EcoRI and Rs16S-R-EcoRI and cloned in the *Eco*RI site of pCPP6529, a mini-Tn7-containing plasmid. The resulting plasmids pCPP6529-ISPC and pTNS2, encoding the TnsABCD site-specific transposition pathway, were co-transformed into *R. pickettii* JCM 5969 by electroporation, as described previously (66). The colonies selected from BG plates containing kanamycin were confirmed by PCR using primers Tn7 check glmS-F/Tn7 check R. The positive recombinants that produced a 1,763-bp PCR band were further confirmed by sequencing the PCR products.

### qPCR assays and standard curve generation

The qPCR reaction system had a total volume of 25 µL, including 400 nM of each primer, 400 nM TaqMan MGB probe, 3 mM MgCl_2_, 200 µM of each dNTP, 5 µL 0.1% bovine serum albumin, 1× PCR buffer, 2.5 U of *Takara Taq* DNA polymerase (Takara, Dalian, China), and 5 µL template DNA. The temperature profile was as follows: an initial denaturation step of 95°C for 5 min, 45 cycles of 95°C for 15 s, and 60°C for 30 s, in an Archimed X6 equipment (Rocgene, Beijing, China). The RSSC16s-F/Rs16S-R primer pair was shared for both strains, while the probes were different, as RSSC-P and RsPC-P, for the target strain and ISPC strain, respectively. Each qPCR reaction was conducted with three technical replicates.

To establish the standard curve, two amplicons amplified using the RSSC16s-F/Rs16S-R primer pair from LMG 9673 and RsPC were cloned into the pMD18-T vector (Takara) individually. The recombinant plasmids pMD18-Rs16S and pMD18-RsPC were verified by Sanger sequencing (Azenta, Suzhou, China), digested by *Eco*RI (Takara), and purified using the Gel Extraction Kit (Omega, USA). The concentrations of recovered DNA were estimated using a micro-spectrophotometer NAS99 (ACTGene, USA). The copy numbers of recombinant plasmids were calculated using the following equation: copies/μL = [6.02 × 10^23^ (copy mol^−1^) × DNA concentration (g/μL)] / [DNA length (bp) × 660 g mol^−1^ bp^−1^].

The linearized recombinant plasmids were 10-fold serially diluted and used to generate standard curves. The PCR efficiencies were calculated using the following equation:

PCR efficiency = 10^(−1/*k*)^ − 1, the parameter *k* was the slope of the standard curve and was calculated automatically by Microsoft Excel 2016.

### Interaction between target DNA and ISPC DNA

To evaluate the interaction between ISPC DNA and target DNA, one of the templates was detected in the reaction, containing different concentrations of the other one as background. Linearized and gel-purified plasmids, pMD18-Rs16S and pMD18-RsPC, were both added to the same PCR mixture. The final concentrations of tested DNA were adjusted to 10^1^–10^4^ or 10^0^–10^3^ copies/μL, and background DNA was adjusted to 10^1^–10^7^ or 10^0^–10^6^ copies/μL, respectively. The PCR sensitivity was evaluated to indicate the interaction between two DNA templates.

### Estimation of REs for *Ralstonia* strains and RsPC in soil samples

To evaluate the REs of LMG 9673 and RsPC in 10 soil samples, 1.70 × 10^4^ CFU of LMG 9673 and 4.26 × 10^4^ CFU of RsPC were added to the same spin column together with 0.25 g of soil with three biological replicates, and the DNA extraction procedures were carried out according to the manufacturer’s instructions. The concentrations of bacterial cultures were determined by plate counting on CPG plates with three technical replicates. For the control groups, soil DNA was extracted without bacterial inoculation. The extracted soil DNA was detected by qPCR as described above.

The RE was calculated using the following equation:


$$\text{RE =}\frac{\text{Detected quantity}}{\text{Inoculated quantity}}\times 100\%$$

The normalized RE of the target strain by RsPC was calculated using the following equation:


$${\text{Normalized RE}}_{\text{target}}\text{ = }\frac{{\text{RE}}_{\text{target}}}{{\text{RE}}_{\text{ISPC}}}$$

The relative accuracy was calculated using the following equation:


$$\text{Relative accuracy =}{\text{log}}_{\text{2}}\text{(}\frac{\text{1}}{\text{RE}}\text{)}$$

Soil samples from Kunming, Chengdu, and Wulanchabu were selected as representative samples and used to investigate the correction effect of different RSSC strains (*R. pseudosolanacearum* LMG 9673, *R. solanacearum* NCPPB 325, and *R. syzygii* LLRS-1) at different concentrations. RsPC (approximately 10^4^ or 10^6^ CFU) was co-extracted with a certain RSSC strain (approximately 10^5^, 10^4^, and 10^3^ CFU) with three biological replicates. The CFU was determined by plate counting on CPG plates for strains LMG 9673 and NCPPB 325 and on CPG plates containing 0.025% ammonium ferric citrate for stain LLRS-1. The detection and quantification methods were as mentioned above.

One-way analysis of variance and the *t*-test were used to analyze significant differences (*P* < 0.05) using IBM SPSS Statistics 24 (IBM Corp., Armonk, NY, USA). The regression analysis and the extra-sum-of-squares F-test (*P* < 0.05) were conducted using GraphPad Prism 9 (GraphPad Software, San Diego, CA, USA). The Ct values of qPCR experiments for evaluation of RE values were reported in Tables S6, S7, and S8.

## SUPPLEMENTAL MATERIAL

Supplemental material spectrum.00210-23-s0001.docx Table S1 to S8 and Fig. S1.

ASM does not own the copyrights to Supplemental Material that may be linked to, or accessed through, an article. The authors have granted ASM a non-exclusive, world-wide license to publish the Supplemental Material files. Please contact the corresponding author directly for reuse.
