# Supplementary material for: Quantitative detection of the Ralstonia solanacearum species complex in soil by qPCR combined with a recombinant internal control strain
Source: Microbiol Spectr. 2023 Nov 15;11(6):e00210-23. doi: 10.1128/spectrum.00210-23 (PMC10715031; doi:10.1128/spectrum.00210-23)
Supplement: Supplemental material — Table S1 to S8 and Fig. S1. [file spectrum.00210-23-s0001.docx]

**Supplementary materials**

Quantitative detection of *Ralstonia solanacearum* species complex in soil by qPCR combined with a recombinant internal control strain

Wei Chen,^a^ Jun-Wei Zhang,^a^ Bi-Xia Qin,^b^ Hui-Ting Xie,^b^ Zhi Zhang,^a^ Xiu-Ze Qiao,^a^ Shan-Kui Li,^a^ Muhammad Asif,^a^ Song Guo,^a^ Li-Xian Cui,^b^ Pei-Pei Wang,^c^ Li-Hong Dong,^c^ Qing-Gang Guo,^c^ Wen-Jun Jiang,^a^ Ping Ma,^c^ Zhen-Yuan Xia,^d^ Can-Hua Lu,^d^# Li-Qun Zhang,^a^#

^a^Ministry of Agriculture and Rural Affairs Key Laboratory of Pest Monitoring and Green Management, College of Plant Protection, China Agricultural University, 2 Yuanmingyuan West Road, Beijing 100193, China

^b^Plant Protection Research Institute, Guangxi Academy of Agricultural Sciences, Guangxi Key Laboratory of Biology for Crop Diseases and Insect Pests, 174 Daxuedong Road, Xixiangtang District, Nanning 530007, China

^c^Institute of Plant Protection, Hebei Academy of Agriculture and Forestry Sciences, Key Laboratory of IPM on Crops in Northern Region of North China, Ministry of Agriculture and Rural Affairs, IPM Innovation Centre of Hebei Province, 437 Dongguan Street, Lianchi District, Baoding 071000, China

^d^Yunnan Academy of Tobacco Agricultural Sciences, 33 Yuantong Road, Wuhua District, Kunming 650021, China

Table S1. Information of 603 *Ralstonia* strains used in this study

| Strain | Origin | Sequencing method | Host | Assembly ID | Assembly level | number of contigs/scaffolds | number of tRNAs | number of rRNAs | number of CDS | taxonomy | Copy number of 16S rDNA per genome |
| --- | --- | --- | --- | --- | --- | --- | --- | --- | --- | --- | --- |
| AU12-08 | Australia: QLD | 454 GS FLX Titanium | *Homo sapiens* | GCF_000442475.1 | contig | - | - | - | - | *R. insidiosa* | - |
| 58_RSOL | USA: WA | Illumina HiSeq | *Homo sapiens* | GCA_001065525.1 | scaffold | - | - | - | - | *R. insidiosa* | - |
| NT80 | Japan; Ehime, Niihama | llumina Solexa | Laurel forest soil | GCA_001485395.1 | contig | - | - | - | - | *R. insidiosa* | - |
| FC1138 | USA: Jessup, MD | PacBio | plant | GCA_001653935.1 | complete genome | - | - | - | - | *R. insidiosa* | - |
| ATCC 49129 | unknown | Illumina | - | GCA_001663855.1 | complete genome | - | - | - | - | *R. insidiosa* | - |
| WCHRI065437 | China: Sichuan, Chengdu | Illumina HiSeq | *Homo sapiens* | GCF_002939035.1 | contig | - | - | - | - | *R. insidiosa* | - |
| WCHRI065162 | China: Sichuan, Chengdu | Illumina HiSeq | *Homo sapiens* | GCF_002939165.1 | contig | - | - | - | - | *R. insidiosa* | - |
| CCUG 46789* | USA | Illumina MiSeq | *Homo sapiens* | GCF_008801405.1 | contig | - | - | - | - | *R. insidiosa* | - |
| 5047 | Brazil: Sao Paulo | Illumina MiSeq | *Homo sapiens* | GCF_012956805.1 | contig | - | - | - | - | *R. insidiosa* | - |
| AU6853 | USA | Illumina | *Homo sapiens* | GCA_019795305.1 | contig | - | - | - | - | *R. insidiosa* | - |
| AU39423 | USA | Illumina | *Homo sapiens* | GCA_019795515.1 | contig | - | - | - | - | *R. insidiosa* | - |
| 58/1 | Russia: Samara | MGI | *Homo sapiens* | GCA_022214855.1 | scaffold | - | - | - | - | *R. insidiosa* | - |
| SN82F48 | USA: San Francisco | Oxford Nanopore; Illumina | *Homo sapiens* | GCA_000954135.2 | complete genome | - | - | - | - | *R. mannitolilytica* | - |
| SN83A39 | USA: San Francisco | Nextera paired-end, Illumina; Nextera mate-pair, Illumina; Oxford nanopore | *Homo sapiens* | GCA_001628775.1 | complete genome | - | - | - | - | *R. mannitolilytica* | - |
| FDAARGOS_410 | unknown | PacBio; Illumina | *Homo sapiens* | GCA_002393485.1 | complete genome | - | - | - | - | *R. mannitolilytica* | - |
| MRY14-0246 | Japan | Illumina HiSeq 2500 | *Homo sapiens* | GCF_000953875.1 | contig | - | - | - | - | *R. mannitolilytica* | - |
| WCHRM065837 | China: Sichuan, Chengdu | Illumina HiSeq | *Homo sapiens* | GCF_002939145.1 | contig | - | - | - | - | *R. mannitolilytica* | - |
| NCTC10894 | unknown | - | - | GCA_900455575.1 | contig | - | - | - | - | *R. mannitolilytica* | - |
| NCTC10893 | United Kingdom: London | - | - | GCA_900455685.1 | contig | - | - | - | - | *R. mannitolilytica* | - |
| NCTC12379 | United Kingdom: London | - | *Homo sapiens* | GCA_900461715.1 | contig | - | - | - | - | *R. mannitolilytica* | - |
| LMG 6866* | United Kingdom | - | - | GCA_905397375.1 | contig | - | - | - | - | *R. mannitolilytica* | - |
| AU11682 | USA | Illumina | *Homo sapiens* | GCF_019042415.1 | contig | - | - | - | - | *R. mannitolilytica* | - |
| WCHRM065694 | China: Sichuan, Chengdu | Illumina HiSeq | *Homo sapiens* | GCF_002939115.1 | contig | - | - | - | - | *R. mannitolilytica* | - |
| Guangzhou-RMAB10 | China: Guangzhou | PacBio | *Homo sapiens* | GCA_011044515.1 | complete genome | - | - | - | - | *R. mannitolilytica* | - |
| HI3842 | USA | Illumina | - | GCA_019795175.1 | contig | - | - | - | - | *R. mannitolilytica* | - |
| GML-Rals1-TR | Turkey: Kayseri | Illumina MiSeq; Oxford Nanopore | *Homo sapiens* | GCF_002863525.1 | chromosome |  |  |  |  | *R. mannitolilytica* | - |
| 3N | USA: Dryden | Illumina MiSeq | - | GCA_013177745.1 | contig | - | - | - | - | *R. pickettii* | - |
| LB_tupeA | Brazil: Amazon State | Illumina HiSeq | - | GCA_013391365.1 | contig | - | - | - | - | *R. pickettii* | - |
| T9CP10 | Portugal | Illumina HiSeq | - | GCA_013413505.1 | contig | - | - | - | - | *R. pickettii* | - |
| s65 | USA: International Space Station | Illumina NextSeq | - | GCA_014141555.1 | scaffold | - | - | - | - | *R. pickettii* | - |
| s64 | USA: International Space Station | Illumina NextSeq | - | GCA_014141585.1 | scaffold | - | - | - | - | *R. pickettii* | - |
| s55 | USA: International Space Station | Illumina NextSeq | - | GCA_014141695.1 | scaffold | - | - | - | - | *R. pickettii* | - |
| s51 | USA: International Space Station | Illumina NextSeq | - | GCA_014141735.1 | scaffold |  |  |  |  | *R. pickettii* | - |
| s49 | USA: International Space Station | Illumina NextSeq | - | GCA_014141745.1 | scaffold | - | - | - | - | *R. pickettii* | - |
| s50 | USA: International Space Station | Illumina NextSeq | - | GCA_014141805.1 | scaffold | - | - | - | - | *R. pickettii* |  |
| s48 | USA: International Space Station | Illumina NextSeq | - | GCA_014141825.1 | scaffold | - | - | - | - | *R. pickettii* | - |
| s46 | USA: International Space Station | Illumina NextSeq | - | GCA_014141845.1 | scaffold | - | - | - | - | *R. pickettii* | - |
| s43 | USA: International Space Station | Illumina NextSeq | - | GCA_014141915.1 | contig | - | - | - | - | *R. pickettii* | - |
| s38 | USA: International Space Station | Illumina NextSeq | - | GCA_014142025.1 | scaffold | - | - | - | - | *R. pickettii* | - |
| s34 | USA: International Space Station | Illumina NextSeq | - | GCA_014142125.1 | contig | - | - | - | - | *R. pickettii* | - |
| s32 | USA: International Space Station | Illumina NextSeq | - | GCA_014142135.1 | scaffold | - | - | - | - | *R. pickettii* | - |
| s30 | USA: International Space Station | Illumina NextSeq | - | GCA_014142165.1 | scaffold | - | - | - | - | *R. pickettii* | - |
| s29 | USA: International Space Station | Illumina NextSeq | - | GCA_014142195.1 | scaffold | - | - | - | - | *R. pickettii* | - |
| s21 | USA: International Space Station | Illumina NextSeq | - | GCA_014142365.1 | scaffold | - | - | - | - | *R. pickettii* | - |
| s19 | USA: International Space Station | Illumina NextSeq | - | GCA_014142405.1 | contig | - | - | - | - | *R. pickettii* | - |
| s8 | USA: International Space Station | Illumina NextSeq | - | GCA_014142625.1 | contig | - | - | - | - | *R. pickettii* | - |
| s7 | USA: International Space Station | Illumina NextSeq | - | GCA_014142645.1 | scaffold | - | - | - | - | *R. pickettii* | - |
| s6 | USA: International Space Station | Illumina NextSeq | - | GCA_014142655.1 | contig | - | - | - | - | *R. pickettii* | - |
| s4 | USA: International Space Station | Illumina NextSeq | - | GCA_014142685.1 | scaffold | - | - | - | - | *R. pickettii* | - |
| s5 | USA: International Space Station | Illumina NextSeq | - | GCA_014142695.1 | contig | - | - | - | - | *R. pickettii* | - |
| s3 | USA: International Space Station | Illumina NextSeq | - | GCA_014142735.1 | contig | - | - | - | - | *R. pickettii* | - |
| K-288 | USA | Illumina HiSeq; PacBio RS | *Homo sapiens* | GCA_016466415.2 | chromosome | - | - | - | - | *R. pickettii* | - |
| B265 | USA | Illumina HiSeq; Oxford Nanopore | *Arachis hypogaea* | GCA_018726805.1 | contig | - | - | - | - | *R. pickettii* | - |
| 093350054-1 | - | Illumina NextSeq 500 | - | GCA_019641695.1 | contig | - | - | - | - | *R. pickettii* | - |
| 153490002-3 | - | Illumina NextSeq 500 | - | GCA_019641775.1 | contig | - | - | - | - | *R. pickettii* | - |
| 093490003-1 | - | Illumina NextSeq 500 | - | GCA_019641795.1 | contig | - | - | - | - | *R. pickettii* | - |
| 153490002-2 | - | Illumina NextSeq 500 | - | GCA_019641815.1 | contig | - | - | - | - | *R. pickettii* | - |
| 151470044-2 | - | Illumina NextSeq 500 | - | GCA_019641895.1 | contig | - | - | - | - | *R. pickettii* | - |
| 151470044-3 | - | Illumina NextSeq 500 | - | GCA_019641905.1 | contig | - | - | - | - | *R. pickettii* | - |
| 092570011-1 | - | Illumina NextSeq 500 | - | GCA_019642025.1 | contig | - | - | - | - | *R. pickettii* | - |
| 140710038-2 | - | Illumina NextSeq 500 | - | GCA_019642175.1 | contig | - | - | - | - | *R. pickettii* | - |
| 092950004-1 | - | Illumina NextSeq 500 | - | GCA_019642275.1 | contig | - | - | - | - | *R. pickettii* | - |
| 133170049-3 | - | Illumina NextSeq 500 | - | GCA_019642295.1 | contig | - | - | - | - | *R. pickettii* | - |
| 132550021-3 | - | Illumina NextSeq 500 | - | GCA_019642335.1 | contig | - | - | - | - | *R. pickettii* | - |
| 132550028-1 | - | Illumina NextSeq 500 | - | GCA_019642395.1 | contig | - | - | - | - | *R. pickettii* | - |
| 133170061-1 | - | Illumina NextSeq 500 | - | GCA_019642455.1 | contig | - | - | - | - | *R. pickettii* | - |
| 123250053-1 | - | Illumina NextSeq 500 | - | GCA_019642515.1 | contig | - | - | - | - | *R. pickettii* | - |
| 092570008-1 | - | Illumina NextSeq 500 | - | GCA_019642555.1 | contig | - | - | - | - | *R. pickettii* | - |
| 121850007-2 | - | Illumina NextSeq 500 | - | GCA_019642675.1 | contig | - | - | - | - | *R. pickettii* | - |
| 113330051-2 | - | Illumina NextSeq 500 | - | GCA_019642735.1 | contig | - | - | - | - | *R. pickettii* | - |
| 112760001-1 | - | Illumina NextSeq 500 | - | GCA_019642775.1 | contig | - | - | - | - | *R. pickettii* | - |
| 112620001-1 | - | Illumina NextSeq 500 | - | GCA_019642795.1 | contig | - | - | - | - | *R. pickettii* | - |
| 092160076-1 | - | Illumina NextSeq 500 | - | GCA_019642805.1 | contig | - | - | - | - | *R. pickettii* | - |
| 112060001-1 | - | Illumina NextSeq 500 | - | GCA_019642855.1 | contig | - | - | - | - | *R. pickettii* | - |
| 110730038-1 | - | Illumina NextSeq 500 | - | GCA_019643015.1 | contig | - | - | - | - | *R. pickettii* | - |
| 110730035-1 | - | Illumina NextSeq 500 | - | GCA_019643075.1 | contig | - | - | - | - | *R. pickettii* | - |
| UNCCL144 | - |  | - | GCA_900099845.1 | scaffold | - | - | - | - | *R. pickettii* | - |
| NCTC11149 | USA: California (State) |  | - | GCA_900455835.1 | contig | - | - | - | - | *R. pickettii* | - |
| MGYG-HGUT-01384 | - |  | - | GCF_902374465.1 | scaffold | - | - | - | - | *R. pickettii* | - |
| NBRC 102503 | - | 454 GS-FLX Titanium; Illumina HiSeq 1000 | - | GCF_001544155.1 | contig | - | - | - | - | *R. pickettii* | - |
| 5_7_47FAA | - | 454 | *Homo sapiens* | GCF_000165085.1 | scaffold | - | - | - | - | *R. pickettii* | - |
| 5_2_56FAA | - | 454; Illumina | *Homo sapiens* | GCF_000227255.2 | scaffold | - | - | - | - | *R. pickettii* | - |
| ATCC 27511* | - | 454; Illumina | - | GCF_000743455.1 | scaffold | - | - | - | - | *R. pickettii* | - |
| H2Cu2 | Portugal | Ion Torrent PGM | - | GCA_001699795.1 | contig | - | - | - | - | *R. pickettii* | - |
| ICMP-8657 | Japan:Yatabe | PacBio | *Oryza sativa* | GCA_002516395.2 | scaffold | - | - | - | - | *R. pickettii* | - |
| PSLESD1 | China: Hangzhou | Illumina NovaSeq | *Homo sapiens* | GCA_009668005.1 | contig | - | - | - | - | *R. pickettii* | - |
| FDAARGOS_1535 | Germany: Braunschweig | Pacbio; Illumina | - | GCA_020341455.1 | chromosome | - | - | - | - | *R. pickettii* | - |
| PSI07 | - | - | - | GCA_000283475.1 | complete genome | 2 | 59 | 9 | 4803 | *R. syzygii* | 3 |
| KACC 10722 | South Korea: Jeju | 454; Illumina MiSeq | *Solanum tuberosum* | GCA_001586135.1 | complete genome | 2 | 57 | 6 | 4744 | *R. syzygii* | 2 |
| A2-HR MARDI | Malaysia: Kuala Kangsar, Perak | PacBio | *Musa nana* | GCA_002012345.1 | chromosome | 2 | 61 | 9 | 4604 | *R. syzygii* | - |
| T51 | South Korea: Boseong | PacBio | *Solanum tuberosum* | GCA_003515145.1 | complete genome | 2 | 62 | 9 | 4686 | *R. syzygii* | 3 |
| T11 | South Korea: Gimhae | PacBio | *Solanum tuberosum* | GCA_003515165.1 | complete genome | 2 | 62 | 9 | 4732 | *R. syzygii* | 3 |
| SL3175 | South Korea: Namjeju | PacBio | *Solanum tuberosum* | GCA_003515185.1 | complete genome | 2 | 61 | 9 | 4794 | *R. syzygii* | 3 |
| T98 | South Korea: Namjeju | PacBio | *Solanum tuberosum* | GCA_003515265.1 | complete genome | 2 | 61 | 9 | 4794 | *R. syzygii* | 3 |
| SL2312 | South Korea: Miryang | PacBio | *Solanum tuberosum* | GCA_003515425.1 | complete genome | 2 | 60 | 9 | 4706 | *R. syzygii* | 3 |
| SL2064 | South Korea: Namjeju | PacBio | *Solanum tuberosum* | GCA_003515445.1 | complete genome | 2 | 62 | 9 | 4753 | *R. syzygii* | 3 |
| T101 | South Korea: Namjeju | PacBio | *Solanum tuberosum* | GCA_003515485.1 | complete genome | 2 | 61 | 9 | 4713 | *R. syzygii* | 3 |
| T95 | South Korea: Namjeju | PacBio | *Solanum tuberosum* | GCA_003515505.1 | complete genome | 2 | 62 | 9 | 4747 | *R. syzygii* | 3 |
| T82 | South Korea: Gimje | PacBio | *Solanum tuberosum* | GCA_003515525.1 | complete genome | 2 | 61 | 9 | 4694 | *R. syzygii* | 3 |
| GMSS_Db01 | Indonesia: Yogyakarta | Oxford Nanopore GridION | *Musa balbisiana* | GCA_016743075.1 | complete genome | 2 | 61 | 9 | 4747 | *R. syzygii* | 3 |
| LLRS-1 | China: Yunnan province | Illumina HiSeq | *Nicotiana tabacum* | GCA_018243215.1 | complete genome | 2 | 63 | 9 | 4891 | *R. syzygii* | 3 |
| NCPPB 3727 | - | PacBio Sequel | *Musa* sp. | GCF_015910595.1 | contig | 4 | 60 | 10 | 4669 | *R. syzygii* | - |
| NCPPB 3219 | Indonesia | PacBio Sequel | *Syzygium aromaticum* | GCF_015910645.1 | contig | 18 | 62 | 9 | 4989 | *R. syzygii* | - |
| NCPPB 3445 | - | PacBio Sequel | *Syzygium aromaticum* | GCA_015910655.1 | contig | 16 | 57 | 8 | 4404 | *R. syzygii* | - |
| T12 | South Korea: Namhae | PacBio | *Solanum tuberosum* | GCA_003515325.1 | complete genome | 2 | 60 | 9 | 4834 | *R. syzygii* | 3 |
| SL3022 | South Korea: Gimhae | PacBio | *Solanum tuberosum* | GCA_003515385.1 | complete genome | 2 | 64 | 9 | 4958 | *R. syzygii* | 3 |
| UQRS280_UW738 | Indonesia | Illumina | *Solanum lycopersicum* | GCA_023075235.1 | contig | 85 | 55 | 3 | 4823 | *R. syzygii* | - |
| CFBP6727_UW736 | Martinique | Illumina | *Heloconia caribea* | GCA_023075255.1 | contig | 97 | 55 | 3 | 4807 | *R. syzygii* | - |
| MAFF301552_UW651 | Japan | Illumina | *Solanum lycopersicum* | GCA_023075435.1 | contig | 60 | 53 | 3 | 4615 | *R. syzygii* | - |
| MAFF301558_UW652 | Japan | Illumina | *Solanum tuberosum* | GCA_023075455.1 | contig | 47 | 54 | 3 | 4683 | *R. syzygii* | - |
| UW258 | Costa Rica | PacBio | *Solanum tuberosum* | GCA_023077135.1 | contig | 6 | 59 | 9 | 4912 | *R. syzygii* | - |
| GMI1000 | China | 454 | - | GCA_000009125.1 | complete genome | 2 | 64 | 12 | 5048 | *R. pseudosolanacearum* | 4 |
| FQY_4 | - | Illumina | - | GCA_000348545.1 | chromosome | 2 | 59 | 12 | 5056 | *R. pseudosolanacearum* | - |
| CMR15 | - | - | - | GCA_000427195.1 | chromosome | 3 | 71 | 12 | 4881 | *R. pseudosolanacearum* | - |
| SD54 | China | 454 | - | GCA_000430925.2 | contig | 165 | 56 | 3 | 5008 | *R. pseudosolanacearum* | - |
| CFBP3059 | China | 454 | - | GCA_001644855.1 | contig | 399 | 62 | 3 | 4749 | *R. pseudosolanacearum* | - |
| P781 | USA: Florida | Illumina HiSeq | *Mandevilla* sp. | GCA_001644865.1 | contig | 298 | 60 | 3 | 4907 | *R. pseudosolanacearum* | - |
| UW757 | Guatemala | Illumina MiSeq | *Osteospermum* sp. | GCA_001645725.1 | scaffold | 222 | 59 | 3 | 4920 | *R. pseudosolanacearum* | - |
| YC40-M | China | PacBio | *Rhizoma kaempferiae* | GCA_001663415.1 | complete genome | 2 | 64 | 12 | 5033 | *R. pseudosolanacearum* | 4 |
| KACC10709 | Korea: Gimcheon | 454_FLX_8K paired end; Illumina_MiSeq_250_paired end | *Solanum tuberosum* | GCA_001708525.1 | complete genome | 2 | 66 | 12 | 4896 | *R. pseudosolanacearum* | 4 |
| PSS1308 | China：Taiwan | Illumina GAIIx | *Solanum tuberosum* | GCA_001870805.1 | contig | 162 | 53 | 3 | 4960 | *R. pseudosolanacearum* | - |
| PSS190 | China：Taiwan | Illumina GAIIx | *Solanum tuberosum* | GCA_001870825.1 | contig | 189 | 56 | 3 | 4820 | *R. pseudosolanacearum* | - |
| OE1-1 | - | 454; solid | Solanum melongena | GCA_001879565.1 | complete genome | 2 | 65 | 12 | 4967 | *R. pseudosolanacearum* | 4 |
| FJAT-1458 | China: Fujian province | PacBio | *Solanum lycopersicum* | GCA_001887535.1 | complete genome | 2 | 67 | 12 | 5324 | *R. pseudosolanacearum* | 4 |
| EP1 | China | Illumina; PacBio | *Solanum melongena* | GCA_001891105.1 | complete genome | 2 | 65 | 12 | 5261 | *R. pseudosolanacearum* | 4 |
| FJAT-91 | China: Fujian | PacBio | - | GCA_002155245.1 | complete genome | 2 | 65 | 12 | 5119 | *R. pseudosolanacearum* | 4 |
| SEPPX05 | China: Poyang County, Shangrao | PacBio; Illumina HiSeq | *Sesamum indicum* | GCA_002162015.1 | complete genome | 2 | 67 | 12 | 5409 | *R. pseudosolanacearum* | 4 |
| CQPS-1 | China:ChongQing | PacBio | *Nicotiana tabacum* | GCA_002220465.1 | complete genome | 2 | 67 | 12 | 5216 | *R. pseudosolanacearum* | 4 |
| RSCM | China: Guangdong | Illumina HiSeq | *Cucurbita maxima* | GCA_002894285.1 | complete genome | 2 | 65 | 12 | 5357 | *R. pseudosolanacearum* | 4 |
| Fm03 | China: Liuzhou | Illumina HiSeq | *Siraitia grosvenorii* | GCA_003256405.1 | contig | 219 | 59 | 4 | 5163 | *R. pseudosolanacearum* | - |
| Bg07 | China: Qinzhou | Illumina HiSeq | *Momordica charantia* | GCA_003256445.1 | scaffold | 184 | 60 | 3 | 4984 | *R. pseudosolanacearum* | - |
| SL3103 | South Korea: Haenam | PacBio | *Solanum tuberosum* | GCA_003515205.1 | complete genome | 2 | 66 | 12 | 4928 | *R. pseudosolanacearum* | 4 |
| SL2330 | South Korea: Namhae | PacBio | *Solanum tuberosum* | GCA_003515225.1 | complete genome | 2 | 65 | 12 | 4823 | *R. pseudosolanacearum* | 4 |
| T117 | South Korea: Goesan | PacBio | *Solanum tuberosum* | GCA_003515245.1 | complete genome | 2 | 68 | 12 | 4988 | *R. pseudosolanacearum* | 4 |
| T78 | South Korea: Gimje | PacBio | *Solanum tuberosum* | GCA_003515285.1 | complete genome | 3 | 68 | 12 | 5389 | *R. pseudosolanacearum* | 4 |
| SL3755 | South Korea: Boseong | pacbio | *Solanum tuberosum* | GCA_003515345.1 | complete genome | 2 | 65 | 12 | 4991 | *R. pseudosolanacearum* | 4 |
| SL3730 | South Korea: Muan | pacbio | *Solanum tuberosum* | GCA_003515365.1 | complete genome | 2 | 66 | 12 | 4983 | *R. pseudosolanacearum* | 4 |
| SL2729 | South Korea: Miryang | PacBio | *Solanum tuberosum* | GCA_003515405.1 | complete genome | 2 | 66 | 12 | 4910 | *R. pseudosolanacearum* | 4 |
| T60 | South Korea: Yeonggwang | PacBio | *Solanum tuberosum* | GCA_003515545.1 | complete genome | 2 | 66 | 12 | 5206 | *R. pseudosolanacearum* | 4 |
| T42 | South Korea: Muan | PacBio | *Solanum tuberosum* | GCA_003515565.1 | complete genome | 2 | 64 | 12 | 4776 | *R. pseudosolanacearum* | 4 |
| SL3882 | South Korea: Haenam | PacBio | *Solanum tuberosum* | GCA_003515585.1 | complete genome | 2 | 66 | 12 | 5206 | *R. pseudosolanacearum* | 4 |
| SL3822 | South Korea: Bukjeju | PacBio | *Solanum tuberosum* | GCA_003515605.1 | complete genome | 2 | 66 | 12 | 5104 | *R. pseudosolanacearum* | 4 |
| SL3300 | South Korea: Namjeju | PacBio | *Solanum tuberosum* | GCA_003515625.1 | complete genome | 2 | 67 | 12 | 5110 | *R. pseudosolanacearum* | 4 |
| P824 | USA: Florida | Illumina HiSeq; PacBio | *Vaccinium corymbosum* | GCA_003576625.1 | chromosome | 2 | 67 | 12 | 4947 | *R. pseudosolanacearum* | - |
| RS 476 | Brazil: Dom Pedro, Maranhao | Illumina HiSeq 2500 | *Solanum lycopersicum* | GCA_003595305.1 | complete genome | 2 | 64 | 12 | 5042 | *R. pseudosolanacearum* | 4 |
| T523 | Philippines: Los Banos | PacBio | *Solanum lycopersicum* | GCA_003595325.1 | chromosome | 2 | 65 | 12 | 4945 | *R. pseudosolanacearum* | - |
| Tg03 | China: Nanning | Illumina HiSeq | *Luffa aegyptiaca* | GCA_003725665.1 | scaffold | 181 | 58 | 2 | 5192 | *R. pseudosolanacearum* | - |
| HA4-1 | China:Hubei,Hongan | PacBio | *Arachis hypogaea* | GCA_003999715.1 | complete genome | 3 | 66 | 12 | 5174 | *R. pseudosolanacearum* | 4 |
| UW386 | Nigeria | PacBio Sequel | - | GCA_006088755.1 | complete genome | 2 | 66 | 12 | 4720 | *R. pseudosolanacearum* | 4 |
| B2 | China:Guiyang and Nanxiong | PacBio Sequel; Illumina HiSeq | - | GCA_011290405.1 | complete genome | 2 | 67 | 12 | 5061 | *R. pseudosolanacearum* | 4 |
| 204 | China:Guiyang and Nanxiong | PacBio Sequel; Illumina HiSeq | - | GCA_011290425.1 | complete genome | 2 | 67 | 12 | 5031 | *R. pseudosolanacearum* | 4 |
| 203 | China:Guiyang and Nanxiong | PacBio Sequel; Illumina HiSeq | - | GCA_011290445.1 | complete genome | 2 | 67 | 12 | 5038 | *R. pseudosolanacearum* | 4 |
| 202 | China:Guiyang and Nanxiong | PacBio Sequel; Illumina HiSeq | - | GCA_011290465.1 | complete genome | 2 | 67 | 12 | 5028 | *R. pseudosolanacearum* | 4 |
| Pe_1 | South Korea: Seosan | Oxford Nanopore GridION | *Capsicum annuum* | GCA_011420365.1 | contig | 5 | 66 | 12 | 5104 | *R. pseudosolanacearum* | - |
| Pe_3 | South Korea: Chungju | Oxford Nanopore GridION; Illumina | *Capsicum annuum* | GCA_012062465.1 | contig | 3 | 66 | 12 | 5109 | *R. pseudosolanacearum* | - |
| Pe_39 | South Korea: Jeongeup | Oxford Nanopore GridION; Illumina | *Capsicum annuum* | GCA_012062495.1 | contig | 4 | 67 | 12 | 5164 | *R. pseudosolanacearum* | - |
| Pe_27 | South Korea: Cheongwon | Oxford Nanopore GridION; Illumina | *Capsicum annuum* | GCA_012062505.1 | contig | 9 | 67 | 12 | 5036 | *R. pseudosolanacearum* | - |
| Pe_57 | South Korea: Cheongsong | Oxford Nanopore GridION; Illumina | *Capsicum annuum* | GCA_012062545.1 | contig | 2 | 62 | 12 | 4954 | *R. pseudosolanacearum* | - |
| Pe_2 | South Korea: Cheongwon | Illumina | *Capsicum annuum* | GCA_012062585.1 | scaffold | 213 | 59 | 3 | 4888 | *R. pseudosolanacearum* | - |
| Pe_13 | South Korea: Imsil | Illumina NextSeq | *Capsicum annuum* | GCA_012062595.1 | scaffold | 248 | 62 | 3 | 5129 | *R. pseudosolanacearum* | - |
| Pe_9 | South Korea: Cheongyang | Illumina NextSeq | *Capsicum annuum* | GCA_012062605.1 | scaffold | 278 | 62 | 3 | 4999 | *R. pseudosolanacearum* | - |
| Pe_15 | South Korea: Imsil | Illumina NextSeq | *Capsicum annuum* | GCA_012062645.1 | scaffold | 237 | 62 | 3 | 5314 | *R. pseudosolanacearum* | - |
| Pe_4 | South Korea: Bukjeju | Illumina NextSeq | *Capsicum annuum* | GCA_012062655.1 | scaffold | 203 | 62 | 3 | 4853 | *R. pseudosolanacearum* | - |
| To_42 | South Korea: Chuncheon | Illumina NextSeq | *Solanum lycopersicum* | GCA_012062685.1 | scaffold | 306 | 62 | 3 | 5082 | *R. pseudosolanacearum* | - |
| To_63 | South Korea: Bonghwa | Illumina NextSeq | *Solanum lycopersicum* | GCA_012062695.1 | scaffold | 208 | 59 | 3 | 4916 | *R. pseudosolanacearum* | - |
| To_53 | South Korea: Hongcheon | Illumina NextSeq | *Solanum lycopersicum* | GCA_012062725.1 | scaffold | 231 | 62 | 3 | 4669 | *R. pseudosolanacearum* | - |
| To_36 | South Korea: Pyeongchang | Illumina NextSeq | *Solanum lycopersicum* | GCA_012062745.1 | scaffold | 252 | 62 | 3 | 5056 | *R. pseudosolanacearum* | - |
| To_28 | South Korea: Yanggu | Illumina NextSeq | *Solanum lycopersicum* | GCA_012062765.1 | scaffold | 235 | 62 | 3 | 4968 | *R. pseudosolanacearum* | - |
| To_22 | South Korea: Cheorwon | Illumina NextSeq | *Solanum lycopersicum* | GCA_012062775.1 | scaffold | 223 | 60 | 3 | 4669 | *R. pseudosolanacearum* | - |
| To_7 | South Korea: Hwacheon | Illumina NextSeq | *Solanum lycopersicum* | GCA_012062785.1 | scaffold | 228 | 59 | 3 | 4959 | *R. pseudosolanacearum* | - |
| To_1 | South Korea: Hoengseong | Illumina NextSeq | *Solanum lycopersicum* | GCA_012062825.1 | scaffold | 235 | 62 | 3 | 5147 | *R. pseudosolanacearum* | - |
| Pe_56 | South Korea: Eumseong | Illumina NextSeq | *Capsicum annuum* | GCA_012271305.1 | scaffold | 225 | 59 | 3 | 4928 | *R. pseudosolanacearum* | - |
| Pe_61 | South Korea: Goesan | Illumina NextSeq | *Capsicum annuum* | GCA_012271315.1 | scaffold | 246 | 59 | 3 | 5023 | *R. pseudosolanacearum* | - |
| Pe_52 | South Korea: Goesan | Illumina NextSeq | *Capsicum annuum* | GCA_012271325.1 | scaffold | 240 | 59 | 3 | 4987 | *R. pseudosolanacearum* | - |
| Pe_51 | South Korea: Taean | Illumina NextSeq | *Capsicum annuum* | GCA_012271355.1 | scaffold | 229 | 60 | 3 | 5041 | *R. pseudosolanacearum* | - |
| Pe_49 | South Korea: Cheongyang | Illumina NextSeq | *Capsicum annuum* | GCA_012271385.1 | scaffold | 223 | 63 | 3 | 4959 | *R. pseudosolanacearum* | - |
| Pe_45 | South Korea: Gongju | Illumina NextSeq | *Capsicum annuum* | GCA_012271415.1 | scaffold | 211 | 63 | 3 | 4962 | *R. pseudosolanacearum* | - |
| Pe_42 | South Korea: Naju | Illumina NextSeq | *Capsicum annuum* | GCA_012271435.1 | scaffold | 272 | 62 | 3 | 5068 | *R. pseudosolanacearum* | - |
| Pe_30 | South Korea: Imsil | Illumina NextSeq | *Capsicum annuum* | GCA_012271445.1 | scaffold | 227 | 63 | 3 | 5187 | *R. pseudosolanacearum* | - |
| Pe_28 | South Korea: Hwaseong | Illumina NextSeq | *Capsicum annuum* | GCA_012271485.1 | scaffold | 247 | 59 | 3 | 5082 | *R. pseudosolanacearum* | - |
| Pe_26 | South Korea: Seosan | Illumina NextSeq | *Capsicum annuum* | GCA_012271495.1 | scaffold | 200 | 59 | 3 | 5035 | *R. pseudosolanacearum* | - |
| Pe_18 | South Korea: Haenam | Illumina NextSeq | *Capsicum annuum* | GCA_012271525.1 | scaffold | 254 | 62 | 3 | 5310 | *R. pseudosolanacearum* | - |
| Pe_24 | South Korea: Gongju | Illumina NextSeq | *Capsicum annuum* | GCA_012271545.1 | scaffold | 253 | 62 | 3 | 5185 | *R. pseudosolanacearum* | - |
| FJAT15304.F50 | China:Agricultural Bio-Resources Research Institute, Fujian Academy of Agricultural Sciences, Fuzhou | Oxford Nanopore | *Solanum lycopersicum* | GCA_013306475.1 | complete genome | 2 | 65 | 12 | 4902 | *R. pseudosolanacearum* | 4 |
| FJAT15252.F50 | China:Agricultural Bio-Resources Research Institute, Fujian Academy of Agricultural Sciences, Fuzhou | Oxford Nanopore | *Solanum lycopersicum* | GCA_013306495.1 | complete genome | 2 | 67 | 12 | 5309 | *R. pseudosolanacearum* | 4 |
| FJAT15252.F1 | China:Agricultural Bio-Resources Research Institute, Fujian Academy of Agricultural Sciences, Fuzhou | Oxford Nanopore | *Solanum lycopersicum* | GCA_013306515.1 | complete genome | 2 | 67 | 12 | 5311 | *R. pseudosolanacearum* | 4 |
| FJAT15249.F50 | China:Agricultural Bio-Resources Research Institute, Fujian Academy of Agricultural Sciences, Fuzhou | Oxford Nanopore | *Solanum lycopersicum* | GCA_013306545.1 | complete genome | 2 | 67 | 12 | 5312 | *R. pseudosolanacearum* | 4 |
| FJAT15249.F1 | China:Agricultural Bio-Resources Research Institute, Fujian Academy of Agricultural Sciences, Fuzhou | Oxford Nanopore | *Solanum lycopersicum* | GCA_013306575.1 | complete genome | 2 | 67 | 12 | 5308 | *R. pseudosolanacearum* | 4 |
| FJAT15244.F50 | China:Agricultural Bio-Resources Research Institute, Fujian Academy of Agricultural Sciences, Fuzhou | Oxford Nanopore | *Solanum lycopersicum* | GCA_013306655.1 | complete genome | 2 | 69 | 12 | 5200 | *R. pseudosolanacearum* | 4 |
| FJAT15244.F1 | China:Agricultural Bio-Resources Research Institute, Fujian Academy of Agricultural Sciences, Fuzhou | Oxford Nanopore | *Solanum lycopersicum* | GCA_013306745.1 | complete genome | 2 | 69 | 12 | 5196 | *R. pseudosolanacearum* | 4 |
| FJAT1463.F50 | China:Agricultural Bio-Resources Research Institute, Fujian Academy of Agricultural Sciences, Fuzhou | Oxford Nanopore | *Solanum lycopersicum* | GCA_013306765.1 | complete genome | 2 | 67 | 12 | 5312 | *R. pseudosolanacearum* | 4 |
| FJAT1458.F50 | China:Agricultural Bio-Resources Research Institute, Fujian Academy of Agricultural Sciences, Fuzhou | Oxford Nanopore | *Solanum lycopersicum* | GCA_013306785.1 | complete genome | 2 | 67 | 12 | 5309 | *R. pseudosolanacearum* | 4 |
| FJAT1463.F1 | China:Agricultural Bio-Resources Research Institute, Fujian Academy of Agricultural Sciences, Fuzhou | Oxford Nanopore | *Solanum lycopersicum* | GCA_013306805.1 | complete genome | 2 | 67 | 12 | 5312 | *R. pseudosolanacearum* | 4 |
| FJAT1458.F1 | China:Agricultural Bio-Resources Research Institute, Fujian Academy of Agricultural Sciences, Fuzhou | Oxford Nanopore | *Solanum lycopersicum* | GCA_013306825.1 | complete genome | 2 | 67 | 12 | 5308 | *R. pseudosolanacearum* | 4 |
| FJAT1452.F50 | China:Agricultural Bio-Resources Research Institute, Fujian Academy of Agricultural Sciences, Fuzhou | Oxford Nanopore | *Solanum lycopersicum* | GCA_013306845.1 | complete genome | 2 | 67 | 12 | 4896 | *R. pseudosolanacearum* | 4 |
| FJAT1452.F1 | China:Agricultural Bio-Resources Research Institute, Fujian Academy of Agricultural Sciences, Fuzhou | Oxford Nanopore | *Solanum lycopersicum* | GCA_013306875.1 | complete genome | 2 | 67 | 12 | 4900 | *R. pseudosolanacearum* | 4 |
| FJAT1303.F8 | China:Agricultural Bio-Resources Research Institute, Fujian Academy of Agricultural Sciences, Fuzhou | Oxford Nanopore | *Solanum lycopersicum* | GCA_013306915.1 | complete genome | 2 | 68 | 12 | 5074 | *R. pseudosolanacearum* | 4 |
| FJAT1303.F50 | China:Agricultural Bio-Resources Research Institute, Fujian Academy of Agricultural Sciences, Fuzhou | Oxford Nanopore | *Solanum lycopersicum* | GCA_013306935.1 | complete genome | 2 | 68 | 12 | 5069 | *R. pseudosolanacearum* | 4 |
| FJAT1303.F1 | China:Agricultural Bio-Resources Research Institute, Fujian Academy of Agricultural Sciences, Fuzhou | Oxford Nanopore | *Solanum lycopersicum* | GCA_013306955.1 | complete genome | 2 | 65 | 12 | 4902 | *R. pseudosolanacearum* | 4 |
| FJAT91-F1 | China:Agricultural Bio-Resources Research Institute, Fujian Academy of Agricultural Sciences, Fuzhou | Oxford Nanopore | *Solanum lycopersicum* | GCA_013375715.1 | complete genome | 2 | 65 | 12 | 5114 | *R. pseudosolanacearum* | 4 |
| FJAT91-F8 | China:Agricultural Bio-Resources Research Institute, Fujian Academy of Agricultural Sciences, Fuzhou | Oxford Nanopore | *Solanum lycopersicum* | GCA_013375735.1 | complete genome | 2 | 65 | 12 | 5128 | *R. pseudosolanacearum* | 4 |
| FJAT15244-F8 | China | nanopore | - | GCA_013704765.1 | complete genome | 2 | 69 | 12 | 5211 | *R. pseudosolanacearum* | 4 |
| YQ | China: Zhejiang, Yueqing | Oxford Nanopore | Casuarina equisetifolia | GCA_014041975.1 | complete genome | 2 | 65 | 12 | 5096 | *R. pseudosolanacearum* | 4 |
| RUN2474 | Madagascar | PacBio Sequel | *Solanum tuberosum* | GCA_014884685.1 | complete genome | 2 | 71 | 12 | 5039 | *R. pseudosolanacearum* | 4 |
| RUN2279 | Madagascar | PacBio Sequel | *Solanum tuberosum* | GCA_014884705.1 | complete genome | 2 | 69 | 12 | 5098 | *R. pseudosolanacearum* | 4 |
| UW763 | Senegal | PacBio Sequel | - | GCA_014884725.1 | complete genome | 2 | 63 | 12 | 4955 | *R. pseudosolanacearum* | 4 |
| MAFF 211471 | - | Illumina MiSeq; MiniON | - | GCA_015097935.0 | complete genome | 2 | 67 | 12 | 5310 | *R. pseudosolanacearum* | 4 |
| MAFF 211491 | - | Illumina MiSeq; MiniON | - | GCA_015098255.1 | complete genome | 2 | 67 | 12 | 4978 | *R. pseudosolanacearum* | 4 |
| MAFF 301560 | - | Illumina MiSeq; MiniON | - | GCA_015098475.1 | complete genome | 2 | 65 | 12 | 4972 | *R. pseudosolanacearum* | 4 |
| MAFF 241647 | - | Illumina MiSeq; MiniON | - | GCA_015098595.1 | complete genome | 2 | 67 | 12 | 5086 | *R. pseudosolanacearum* | 4 |
| MAFF 241648 | - | Illumina MiSeq; MiniON | - | GCA_015098755.1 | complete genome | 2 | 67 | 12 | 5174 | *R. pseudosolanacearum* | 4 |
| MAFF 211472 | - | Illumina NovaSeq 6000; PacBio | *Zingiber officinale* | GCA_015698345.1 | complete genome | 2 | 66 | 12 | 5312 | *R. pseudosolanacearum* | 4 |
| MAFF 211479 | - | Illumina NovaSeq 6000; PacBio | *Zingiber officinale* | GCA_015698365.1 | complete genome | 2 | 71 | 12 | 5152 | *R. pseudosolanacearum* | 4 |
| MAFF 311693 | - | Illumina NovaSeq 6000; PacBio | *Curcuma aromatica* | GCA_015698385.1 | complete genome | 3 | 67 | 12 | 5182 | *R. pseudosolanacearum* | 4 |
| RS | China: Yunnan province | Illumina HiSeq | tobacco | GCA_018243235.1 | complete genome | 2 | 65 | 12 | 4768 | *R. pseudosolanacearum* | 4 |
| SY1 | China: Guangdong | PacBio Sequel; Illumina | *Pogostemon cablin* | GCA_018540425.1 | complete genome | 2 | 65 | 12 | 5040 | *R. pseudosolanacearum* | 4 |
| Rs-SY1 | China:Hainan | PacBio RSII |  | GCA_018731965.1 | complete genome | 2 | 65 | 12 | 5019 | *R. pseudosolanacearum* | 4 |
| RS24 | China: Guangdong Province | PacBio; Illumina NovaSeq PE150 | *Solanum muricatum* | GCA_018733955.1 | complete genome | 3 | 64 | 12 | 5323 | *R. pseudosolanacearum* | 4 |
| Rs-T02 | China: Nanning | Illumina HiSeq | *Solanum lycopersicum* | GCF_001484095.1 | scaffold | 169 | 59 | 2 | 5056 | *R. pseudosolanacearum* | - |
| CaRs-Mep | India:Wyanad Meppadi | Ion Torent PGM; Illumina NextSeq 500 | *Amomum cardamon* | GCF_001855495.2 | contig | 13 | 58 | 4 | 4845 | *R. pseudosolanacearum* | - |
| UTT-25 | India: Uttarakhand, foot hills of Haldwani, Nainital | Illumina MiSeq | *Solanum lycopersicum* | GCF_002930085.2 | contig | 1058 | 69 | 16 | 5252 | *R. pseudosolanacearum* | - |
| FJAT91.F50 | China:Agricultural Bio-Resources Research Institute, Fujian Academy of Agricultural Sciences, Fuzhou | Oxford Nanopore | *Solanum lycopersicum* | GCF_013306015.1 | complete genome | 2 | 65 | 12 | 5110 | *R. pseudosolanacearum* | 4 |
| FJAT454.F1 | China:Agricultural Bio-Resources Research Institute, Fujian Academy of Agricultural Sciences, Fuzhou | Oxford Nanopore | *Solanum lycopersicum* | GCF_013306095.1 | complete genome | 2 | 66 | 12 | 5255 | *R. pseudosolanacearum* | 4 |
| FJAT448.F50 | China:Agricultural Bio-Resources Research Institute, Fujian Academy of Agricultural Sciences, Fuzhou | Oxford Nanopore | *Solanum lycopersicum* | GCF_013306155.1 | complete genome | 2 | 66 | 12 | 5270 | *R. pseudosolanacearum* | 4 |
| FJAT448.F1 | China:Agricultural Bio-Resources Research Institute, Fujian Academy of Agricultural Sciences, Fuzhou | Oxford Nanopore | *Solanum lycopersicum* | GCF_013306205.1 | complete genome | 2 | 66 | 12 | 5258 | *R. pseudosolanacearum* | 4 |
| FJAT445.F50 | China:Agricultural Bio-Resources Research Institute, Fujian Academy of Agricultural Sciences, Fuzhou | Oxford Nanopore | *Solanum lycopersicum* | GCF_013306235.1 | complete genome | 2 | 67 | 12 | 4912 | *R. pseudosolanacearum* | 4 |
| FJAT445.F1 | China:Agricultural Bio-Resources Research Institute, Fujian Academy of Agricultural Sciences, Fuzhou | Oxford Nanopore | *Solanum lycopersicum* | GCF_013306255.1 | complete genome | 2 | 67 | 12 | 4896 | *R. pseudosolanacearum* | 4 |
| FJAT442.F50 | China:Agricultural Bio-Resources Research Institute, Fujian Academy of Agricultural Sciences, Fuzhou | Oxford Nanopore | *Solanum lycopersicum* | GCF_013306275.1 | complete genome | 2 | 67 | 12 | 4898 | *R. pseudosolanacearum* | 4 |
| FJAT442.F1 | China:Agricultural Bio-Resources Research Institute, Fujian Academy of Agricultural Sciences, Fuzhou | Oxford Nanopore | *Solanum lycopersicum* | GCF_013306295.1 | complete genome | 2 | 67 | 12 | 4898 | *R. pseudosolanacearum* | 4 |
| FJAT15353.F8 | China:Agricultural Bio-Resources Research Institute, Fujian Academy of Agricultural Sciences, Fuzhou | Oxford Nanopore | *Solanum lycopersicum* | GCF_013306315.1 | complete genome | 2 | 68 | 12 | 5074 | *R. pseudosolanacearum* | 4 |
| FJAT15353.F50 | China:Agricultural Bio-Resources Research Institute, Fujian Academy of Agricultural Sciences, Fuzhou | Oxford Nanopore | *Solanum lycopersicum* | GCF_013306335.1 | complete genome | 2 | 68 | 12 | 5070 | *R. pseudosolanacearum* | 4 |
| FJAT15353.F1 | China:Agricultural Bio-Resources Research Institute, Fujian Academy of Agricultural Sciences, Fuzhou | Oxford Nanopore | *Solanum lycopersicum* | GCF_013306355.1 | complete genome | 2 | 68 | 12 | 5072 | *R. pseudosolanacearum* | 4 |
| FJAT15340.F50 | China:Agricultural Bio-Resources Research Institute, Fujian Academy of Agricultural Sciences, Fuzhou | Oxford Nanopore | *Solanum lycopersicum* | GCF_013306375.1 | complete genome | 2 | 65 | 12 | 4904 | *R. pseudosolanacearum* | 4 |
| FJAT15340.F6 | China:Agricultural Bio-Resources Research Institute, Fujian Academy of Agricultural Sciences, Fuzhou | Oxford Nanopore | *Solanum lycopersicum* | GCF_013306395.1 | complete genome | 2 | 65 | 12 | 4896 | *R. pseudosolanacearum* | 4 |
| FJAT15340.F1 | China:Agricultural Bio-Resources Research Institute, Fujian Academy of Agricultural Sciences, Fuzhou | Oxford Nanopore | *Solanum lycopersicum* | GCF_013306415.1 | complete genome | 2 | 65 | 12 | 4893 | *R. pseudosolanacearum* | 4 |
| FJAT15304.F6 | China:Agricultural Bio-Resources Research Institute, Fujian Academy of Agricultural Sciences, Fuzhou | Oxford Nanopore | *Solanum lycopersicum* | GCF_013306435.1 | complete genome | 2 | 65 | 12 | 4893 | *R. pseudosolanacearum* | 4 |
| FJAT15304.F1 | China:Agricultural Bio-Resources Research Institute, Fujian Academy of Agricultural Sciences, Fuzhou | Oxford Nanopore | *Solanum lycopersicum* | GCF_013306455.1 | complete genome | 2 | 65 | 12 | 4893 | *R. pseudosolanacearum* | 4 |
| FJAT454.F50-1 | China:Agricultural Bio-Resources Research Institute, Fujian Academy of Agricultural Sciences, Fuzhou | Oxford Nanopore | *Solanum lycopersicum* | GCF_014490805.1 | complete genome | 2 | 66 | 12 | 5342 | *R. pseudosolanacearum* | 4 |
| NCPPB 4029 | Sri Lanka | PacBio Sequel | *Solanum tuberosum* | GCF_015910515.1 | contig | 20 | 73 | 14 | 5311 | *R. pseudosolanacearum* | - |
| NCPPB 253 | Mauritius | PacBio Sequel | *Casuarina equisetifolia* | GCF_015910695.1 | contig | 12 | 69 | 12 | 5038 | *R. pseudosolanacearum* | - |
| NCPPB 216 | - | PacBio Sequel | - | GCF_015910735.1 | contig | 22 | 68 | 11 | 4843 | *R. pseudosolanacearum* | - |
| PD 4138 | Netherlands | Illumina HiSeq | *Curcuma longa* | GCF_015911375.1 | contig | 439 | 62 | 3 | 5130 | *R. pseudosolanacearum* | - |
| PD 3634 | Kenya | Illumina HiSeq | *Solanum tuberosum* | GCF_015911495.1 | contig | 389 | 61 | 3 | 4943 | *R. pseudosolanacearum* | - |
| PD 3570 | Venezuela | Illumina HiSeq | *Curcuma longa* | GCF_015911505.1 | contig | 271 | 63 | 3 | 5067 | *R. pseudosolanacearum* | - |
| PD:3205 | Philippines | Illumina HiSeq | *Anthurium andraeanum* | GCF_015911615.1 | contig | 266 | 61 | 3 | 4877 | *R. pseudosolanacearum* | - |
| PD:3196 | Netherlands | Illumina HiSeq | *Curcuma longa* | GCF_015911645.1 | contig | 649 | 61 | 3 | 5137 | *R. pseudosolanacearum* | - |
| PD:7123 | - | PacBio Sequel | *Rosa* sp. | GCF_015911755.1 | contig | 11 | 71 | 16 | 5113 | *R. pseudosolanacearum* | - |
| PD:1256 | France | Illumina HiSeq | *Solanum melongena* | GCF_015912055.1 | contig | 324 | 60 | 3 | 4902 | *R. pseudosolanacearum* | - |
| PD:1419 | - | Illumina HiSeq | *Solanum tuberosum* | GCF_015912145.1 | contig | 317 | 61 | 3 | 5024 | *R. pseudosolanacearum* | - |
| NCPPB:1579 | - | Illumina HiSeq | *Zingiber officinale* | GCF_015912175.1 | contig | 299 | 59 | 3 | 5092 | *R. pseudosolanacearum* | - |
| PD:1255 | France | Illumina HiSeq | *Anthurium andraeanum* | GCF_015912195.1 | contig | 456 | 61 | 3 | 4981 | *R. pseudosolanacearum* | - |
| NCPPB:790 | Costa Rica | Illumina HiSeq | *Physalis angulata* | GCF_015912205.1 | contig | 475 | 58 | 3 | 4976 | *R. pseudosolanacearum* | - |
| Lallmahomed 66 | - | Illumina HiSeq | *Arachis hypogaea* | GCF_015912275.1 | contig | 450 | 60 | 3 | 4983 | *R. pseudosolanacearum* | - |
| Lallmahomed 59 | - | Illumina HiSeq | *Casuarina equisetifolia* | GCF_015912285.1 | contig | 459 | 62 | 3 | 4974 | *R. pseudosolanacearum* | - |
| Lallmahomed 30 | - | Illumina HiSeq | *Solanum lycopersicum* | GCF_015912315.1 | contig | 386 | 61 | 3 | 4949 | *R. pseudosolanacearum* | - |
| Lallmahomed 16 | - | Illumina HiSeq | *Solanum melongena* | GCF_015912335.1 | contig | 453 | 61 | 3 | 5028 | *R. pseudosolanacearum* | - |
| Lallmahomed 54 | - | Illumina HiSeq | *Solanum nigrum* | GCF_015912355.1 | contig | 373 | 60 | 3 | 4960 | *R. pseudosolanacearum* | - |
| Lallmahomed 7 | - | Illumina HiSeq | *Nicotiana tabacum* | GCF_015912375.1 | contig | 368 | 61 | 3 | 4997 | *R. pseudosolanacearum* | - |
| Lallmahomed 13 | - | Illumina HiSeq | *Arachis hypogaea* | GCF_015912385.1 | contig | 487 | 62 | 3 | 4961 | *R. pseudosolanacearum* | - |
| 362200 | China:Fujian | PacBio RSII | *Arachis hypogaea* | GCF_015999365.1 | complete genome | 3 | 69 | 12 | 5041 | *R. pseudosolanacearum* | 4 |
| RD15 | China:Taiwan | Illumina GAIIx | *Solanum tuberosum* | GCF_001854265.1 | scaffold | 139 | 55 | 3 | 4713 | *R. pseudosolanacearum* | - |
| PSS216 | China:Taiwan | Illumina GAII | *Solanum tuberosum* | GCF_001876975.1 | scaffold | 224 | 54 | 3 | 5154 | *R. pseudosolanacearum* | - |
| PSS4 | China:Taiwan | Illumina GAII | *Solanum tuberosum* | GCF_001876985.1 | scaffold | 2 | 63 | 12 | 5105 | *R. pseudosolanacearum* | - |
| LMG 9673* | - | Illumina HiSeq | - | GCA_024925465.1 | complete | 2 | 72 | 12 | 5000 | *R. pseudosolanacearum* | - |
| Lallmahomed 72 | - | Pelargonium capitatum | *Arachis hypogaea* | GCF_015912245.1 | contig | 409 | 60 | 3 | 5004 | *R. pseudosolanacearum* | - |
| RS10 | China:Shandong | Oxford Nanopore | *Nicotiana tabacum* | GCA_018861195.1 | complete genome | 2 | 63 | 12 | 5356 | *R. pseudosolanacearum* | 4 |
| T2C-Rasto | Viet Nam:An Giang | Illumina MiniSeq | *Cucumis sativus* | GCA_019737115.1 | contig | 183 | 59 | 3 | 4891 | *R. pseudosolanacearum* | - |
| FJ1003 | - | Illumina HiSeq | *Nicotiana tabacum* | GCA_020923475.1 | complete genome | 3 | 65 | 12 | 5119 | *R. pseudosolanacearum* | 4 |
| P380 | China: Yunnan | Illumina HiSeq | *Solanum lycopersicum* | GCA_021462455.1 | contig | 162 | 60 | 3 | 5047 | *R. pseudosolanacearum* | - |
| SL1931 | - | Illumina HiSeq | *Morus* sp. | GCF_022625045.1 | complete genome | 4 | 67 | 12 | 5156 | *R. pseudosolanacearum* | 4 |
| T25_UW811 | Togo | Illumina | *Solanum lycopersicum* | GCA_023074975.1 | contig | 206 | 56 | 3 | 5204 | *R. pseudosolanacearum* | - |
| UW81 | Colombia | PacBio | - | GCA_023075015.1 | contig | 4 | 63 | 12 | 5046 | *R. pseudosolanacearum* | - |
| B17_UW800 | Benin | Illumina | - | GCA_023075035.1 | contig | 788 | 54 | 3 | 5007 | *R. pseudosolanacearum* | - |
| B9_UW794 | Benin | Illumina | - | GCA_023075075.1 | contig | 234 | 55 | 3 | 5093 | *R. pseudosolanacearum* | - |
| B12_UW797 | Benin | Illumina | - | GCA_023075095.1 | contig | 214 | 59 | 3 | 5243 | *R. pseudosolanacearum* | - |
| RUN2587_UW776 | Madagascar | Illumina | - | GCA_023075115.1 | contig | 339 | 63 | 3 | 4833 | *R. pseudosolanacearum* | - |
| UQRS637_UW745 | - | Illumina | - | GCA_023075155.1 | contig | 210 | 55 | 3 | 4967 | *R. pseudosolanacearum* | - |
| UW74 | Sri Lanka | Illumina | - | GCA_023075215.1 | contig | 235 | 58 | 3 | 5047 | *R. pseudosolanacearum* | - |
| DGBBC1138_UW685 | Guinea | Illumina | - | GCA_023075375.1 | contig | 85 | 62 | 3 | 4634 | *R. pseudosolanacearum* | - |
| NCPPB332_UW654 | Zimbabwe | Illumina | - | GCA_023075415.1 | contig | 181 | 58 | 3 | 4764 | *R. pseudosolanacearum* | - |
| CIP365_UW642 | Philippines | Illumina | - | GCA_023075535.1 | contig | 256 | 59 | 3 | 4880 | *R. pseudosolanacearum* | - |
| UW613 | Guatemala | Illumina | - | GCA_023075595.1 | contig | 173 | 55 | 3 | 4838 | *R. pseudosolanacearum* | - |
| UW604 | Guatemala | Illumina | - | GCA_023075615.1 | contig | 155 | 53 | 3 | 4982 | *R. pseudosolanacearum* | - |
| Rs123_UW585 | USA | Illumina | - | GCA_023075675.1 | contig | 227 | 59 | 3 | 5102 | *R. pseudosolanacearum* | - |
| CIP266_UW505 | Indonesia | Illumina | - | GCA_023075875.1 | contig | 152 | 55 | 3 | 4969 | *R. pseudosolanacearum* | - |
| CIP264_UW503 | Indonesia | Illumina | - | GCA_023075905.1 | contig | 316 | 56 | 3 | 4612 | *R. pseudosolanacearum* | - |
| CIP296_UW472 | Nigeria | Illumina | - | GCA_023076195.1 | contig | 134 | 66 | 3 | 4677 | *R. pseudosolanacearum* | - |
| O12BS_UW407 | Australia | Illumina | - | GCA_023076615.1 | contig | 135 | 56 | 3 | 4955 | *R. pseudosolanacearum* | - |
| UW393 | South Africa | Illumina | - | GCA_023076675.1 | contig | 162 | 55 | 3 | 5039 | *R. pseudosolanacearum* | - |
| POPS1_UW379 | China | Illumina | - | GCA_023076705.1 | contig | 265 | 59 | 3 | 5107 | *R. pseudosolanacearum* | - |
| UW299 | Brazil | PacBio | - | GCA_023076895.1 | contig | 4 | 65 | 12 | 5161 | *R. pseudosolanacearum* | - |
| UW298 | Philippines | Illumina | - | GCA_023076935.1 | contig | 207 | 59 | 3 | 4952 | *R. pseudosolanacearum* | - |
| UW296 | Sri Lanka | Illumina | - | GCA_023076985.1 | contig | 194 | 64 | 3 | 4964 | *R. pseudosolanacearum* | - |
| UW198 | Philippines | Illumina | - | GCA_023077395.1 | contig | 196 | 57 | 3 | 4958 | *R. pseudosolanacearum* | - |
| UW193 | Peru | PacBio | - | GCA_023077445.1 | contig | 4 | 64 | 12 | 5170 | *R. pseudosolanacearum* | - |
| Pt01 | China | Illumina HiSeq | *Solanum tuberosum* | GCA_023130685.1 | scaffold | 188 | 61 | 3 | 4900 | *R. pseudosolanacearum* | - |
| Tb04 | China | Illumina HiSeq | *Nicotiana tabacum* | GCA_023130705.1 | scaffold | 93 | 64 | 3 | 5197 | *R. pseudosolanacearum* | - |
| Pp26 | China | Illumina HiSeq | *Capsicum annuum* | GCA_023130735.1 | scaffold | 146 | 59 | 3 | 5055 | *R. pseudosolanacearum* | - |
| Pn33 | China | Illumina HiSeq | *Arachis hypogaea* | GCA_023130755.1 | scaffold | 114 | 62 | 3 | 4927 | *R. pseudosolanacearum* | - |
| Gg01 | China | Illumina HiSeq | *Zingiber officinale* | GCA_023130765.1 | scaffold | 184 | 60 | 3 | 5097 | *R. pseudosolanacearum* | - |
| Ec03 | China | Illumina HiSeq | *Eucalyptus robusta* | GCA_023130775.1 | contig | 141 | 58 | 3 | 4963 | *R. pseudosolanacearum* | - |
| Ep08 | China | Illumina HiSeq | *Solanum melongena* | GCA_023130815.1 | scaffold | 175 | 60 | 3 | 4876 | *R. pseudosolanacearum* | - |
| Cm01 | China | Illumina HiSeq | *Chrysanthemum* sp. | GCA_023130835.1 | scaffold | 74 | 60 | 3 | 4925 | *R. pseudosolanacearum* | - |
| PeaFJ1 | China | PacBio Sequel | *Arachis hypogaea* | GCA_023518395.1 | complete genome | 2 | 68 | 12 | 4993 | *R. pseudosolanacearum* | 4 |
| Y45 | - | Illumina GA IIx | - | GCA_000223115.2 | scaffold | 499 | 60 | 6 | 4985 | *R. pseudosolanacearum* | - |
| Rs-09-161 | India | Illumina HiSeq | - | GCA_000671335.1 | chromosome | 75 | 64 | 3 | 4982 | *R. pseudosolanacearum* | - |
| YC45 | China | PacBio | *Zingiber officinale* | GCA_001267515.1 | complete genome | 2 | 52 | 5 | 4958 | *R. pseudosolanacearum* | 2 |
| Cq01 | China: Nanning | Illumina HiSeq | *Benincasa hispida* | GCA_003256425.1 | scaffold | 195 | 59 | 2 | 5149 | *R. pseudosolanacearum* | - |
| T25 | South Korea: Miryang | PacBio | *Solanum tuberosum* | GCA_003515305.1 | complete genome | 2 | 64 | 12 | 5346 | *R. pseudosolanacearum* | 4 |
| T110 | South Korea: Bukjeju | PacBio | *Solanum tuberosum* | GCA_003515465.1 | complete genome | 2 | 65 | 12 | 6273 | *R. pseudosolanacearum* | 4 |
| VT0801 | Japan: Tsu City, Mie Prefecture | IonTorrent | *Solanum lycopersicum* | GCA_008000395.1 | contig | 1200 | 54 | 2 | 5427 | *R. pseudosolanacearum* | - |
| 10180 | Philippines | IonTorrent | *Solanum lycopersicum* | GCA_008271775.1 | contig | 888 | 54 | 3 | 5244 | *R. pseudosolanacearum* | - |
| 10154 | Philippines | IonTorrent | *Solanum lycopersicum* | GCA_008271845.1 | contig | 509 | 55 | 3 | 4935 | *R. pseudosolanacearum* | - |
| 10179 | Philippines | IonTorrent | *Solanum lycopersicum* | GCA_008271855.1 | contig | 785 | 55 | 3 | 5100 | *R. pseudosolanacearum* | - |
| UW700 | USA:Virginia | PacBio Sequel | *Solanum lycopersicum* | GCA_002251605.3 | contig | 11 | 61 | 10 | 4844 | *R. solanacearum* | - |
| MolK2 | - | - | - | GCF_000212635.3 | contig | 349 | 43 | 4 | 4994 | *R. solanacearum* | - |
| Po82 | - | - | - | GCF_000215325.1 | complete genome | 2 | 61 | 9 | 4739 | *R. solanacearum* | 3 |
| CFIA906 | India | Illumina MiSeq | *Solanum tuberosum* | GCF_000710135.2 | scaffold | 165 | 61 | 3 | 4558 | *R. solanacearum* | - |
| NCPPB909 | Egypt | Illumina HiSeq | *Solanum tuberosum* | GCF_000710695.1 | scaffold | 215 | 61 | 3 | 4531 | *R. solanacearum* | - |
| 23-10BR | Brazil | 454 | - | GCF_000749995.1 | contig | 94 | 60 | 3 | 4918 | *R. solanacearum* | - |
| NCPPB 282 | Colombia | 454 | *Solanum tuberosum* | GCF_000750575.1 | contig | 181 | 59 | 3 | 4608 | *R. solanacearum* | - |
| POPS2 | China | 454 | - | GCF_000750585.1 | contig | 703 | 56 | 3 | 4902 | *R. solanacearum* | - |
| B50 | - | - | - | GCF_000825785.1 | scaffold | 1 | 56 | 3 | 4979 | *R. solanacearum* | - |
| UW179 | - | - | - | GCF_000825805.1 | scaffold | 1 | 50 | 3 | 4775 | *R. solanacearum* | - |
| CIP417 | - | - | - | GCF_000825825.1 | scaffold | 1 | 56 | 3 | 4840 | *R. solanacearum* | - |
| Grenada 9-1 | - | - | - | GCF_000825845.1 | scaffold | 1 | 55 | 3 | 4804 | *R. solanacearum* | - |
| CFBP1416 | - | - | - | GCF_000825925.1 | scaffold | 1 | 56 | 3 | 5122 | *R. solanacearum* | - |
| UY031 | Uruguay | PacBio | *Solanum commersonii* | GCF_001299555.1 | complete genome | 2 | 65 | 9 | 4684 | *R. solanacearum* | 3 |
| CFBP7014 | - | - | - | GCF_001373255.1 | scaffold | 1 | 54 | 3 | 4922 | *R. solanacearum* | - |
| IBSBF1900 | - | - | - | GCF_001373275.1 | scaffold | 1 | 55 | 3 | 5141 | *R. solanacearum* | - |
| RS2 | - | - | - | GCF_001373295.1 | scaffold | 1 | 59 | 3 | 4193 | *R. solanacearum* | - |
| UW181 | - | - | - | GCF_001373315.1 | scaffold | 1 | 55 | 3 | 4736 | *R. solanacearum* | - |
| CFBP3858 | - | - | - | GCF_001373335.1 | scaffold | 1 | 61 | 3 | 5112 | *R. solanacearum* | - |
| UW163 | Peru: Nauta | PacBio RSII corrected with Illumina MiSeq | - | GCF_001587135.1 | complete genome | 4 | 61 | 9 | 4892 | *R. solanacearum* | 3 |
| IBSBF1503 | Brazil | PacBio RSII corrected with Illumina MiSeq | *Cucumis sativus* | GCF_001587155.1 | complete genome | 2 | 62 | 9 | 4757 | *R. solanacearum* | 3 |
| CIP120 | Peru | Illumina HiSeq | *Solanum tuberosum* | GCF_001644795.1 | contig | 145 | 60 | 3 | 4947 | *R. solanacearum* | - |
| P597 | USA: Mid-Florida | Illumina HiSeq | *Solanum lycopersicum* | GCF_001644805.1 | contig | 530 | 59 | 3 | 5000 | *R. solanacearum* | - |
| CFBP6783 | Martinique | Illumina HiSeq | *Heliconia* sp. | GCF_001644815.1 | contig | 178 | 58 | 3 | 4843 | *R. solanacearum* | - |
| UW491 | Colombia | Illumina MiSeq | *Solanum tuberosum* | GCF_001696845.1 | contig | 118 | 61 | 3 | 4580 | *R. solanacearum* | - |
| UW365 | China | Illumina MiSeq | *Solanum tuberosum* | GCF_001696865.1 | contig | 136 | 61 | 3 | 4522 | *R. solanacearum* | - |
| UW551 | Kenya | PacBio | *Geranium* sp. | GCF_002251655.1 | contig | 130 | 61 | 3 | 4514 | *R. solanacearum* | - |
| K60 | USA:North Carolina | PacBio | *Solanum lycopersicum* | GCF_002251695.1 | contig | 347 | 51 | 3 | 3395 | *R. solanacearum* | - |
| RS 488 | Brazil: Grandes Rios, Parana | Illumina HiSeq 2500 | *Solanum lycopersicum* | GCF_002501565.1 | complete genome | 2 | 65 | 9 | 4685 | *R. solanacearum* | 3 |
| RS 489 | Brazil: Borrazopolis, Parana | Illumina HiSeq 2500 | *Solanum lycopersicum* | GCF_002549815.1 | complete genome | 2 | 60 | 9 | 4687 | *R. solanacearum* | 3 |
| GEO_304 | Georgia | Illumina | *Solanum tuberosum* | GCF_002894775.1 | contig | 110 | 58 | 3 | 4427 | *R. solanacearum* | - |
| GEO_81 | Georgia | Illumina | *Solanum tuberosum* | GCF_002894785.1 | contig | 135 | 58 | 3 | 4372 | *R. solanacearum* | - |
| GEO_230 | Georgia | Illumina | *Solanum tuberosum* | GCF_002894795.1 | contig | 125 | 41 | 3 | 4150 | *R. solanacearum* | - |
| GEO_55 | Georgia | Illumina | *Solanum tuberosum* | GCF_002894845.1 | contig | 168 | 57 | 3 | 4487 | *R. solanacearum* | - |
| IBSBF 2570 | Brazil: Sergipe, Northeastern region of Brazil | Illumina MiSeq-2500 Platform | Musa sp. | GCF_003590585.1 | complete genome | 2 | 60 | 9 | 4761 | *R. solanacearum* | 3 |
| IBSBF 2571 | Brazil: Sergipe, Northeastern region of Brazil | Illumina MiSeq | Musa sp. | GCF_003590605.1 | complete genome | 2 | 60 | 9 | 4771 | *R. solanacearum* | 3 |
| SFC | Brazil: Sergipe, Northeastern region of Brazil | Illumina MiSeq-2500 Platform | Musa sp. | GCF_003590625.1 | complete genome | 2 | 61 | 9 | 4749 | *R. solanacearum* | 3 |
| CRMRs218 | Brazil: Camocim de Sao Felix, Pernambuco | Illumina HiSeq | *Solanum melongena* | GCF_003612975.2 | chromosome | 2 | 62 | 9 | 4502 | *R. solanacearum* | - |
| UA-1612 | Colombia: North caribbean, Magdalena | Illumina MiSeq | *Musa nana* | GCF_003860665.1 | chromosome | 1 | 56 | 3 | 4454 | *R. solanacearum* | - |
| UA-1611 | Colombia: North caribbean, Magdalena | Illumina MiSeq | *Musa nana* | GCF_003860685.1 | chromosome | 1 | 61 | 4 | 4570 | *R. solanacearum* | - |
| UA-1591 | Colombia: North caribbean, Magdalena | Illumina MiSeq | *Musa nana* | GCF_003860705.1 | chromosome | 1 | 57 | 3 | 4690 | *R. solanacearum* | - |
| UA-1579 | Colombia: North caribbean, Magdalena | Illumina MiSeq | *Musa nana* | GCF_003860725.1 | chromosome | 1 | 56 | 3 | 4455 | *R. solanacearum* | - |
| UA-1617 | Colombia: North caribbean, Magdalena | Illumina MiSeq | *Musa nana* | GCF_003860745.1 | chromosome | 1 | 58 | 3 | 4684 | *R. solanacearum* | - |
| UA-1609 | Colombia: North caribbean, Magdalena | Illumina MiSeq | *Musa nana* | GCF_003860765.1 | chromosome | 1 | 56 | 3 | 4456 | *R. solanacearum* | - |
| UW848 | USA | Illumina iSeq | *Geranium* sp. | GCF_013359535.1 | contig | 160 | 61 | 3 | 4521 | *R. solanacearum* | - |
| CCRMRs304 | Brazil: Fonte Boa, Amazonas | Illumina MiSeq | *Musa nana* | GCF_014210335.1 | scaffold | 2 | 53 | 3 | 4545 | *R. solanacearum* | - |
| CCRMRsB7 | Brazil: Anama, Amazonas | Illumina MiSeq | *Musa nana* | GCF_014210345.1 | scaffold | 2 | 63 | 3 | 4986 | *R. solanacearum* | - |
| CCRMRs287 | Brazil: Anama, Amazonas | Illumina MiSeq | *Musa nana* | GCF_014210375.1 | scaffold | 2 | 57 | 3 | 4649 | *R. solanacearum* | - |
| CCRMRs277 | Brazil: Anama, Amazonas | Illumina MiSeq | *Musa nana* | GCF_014210395.1 | scaffold | 2 | 53 | 3 | 4578 | *R. solanacearum* | - |
| Rs5 | USA: Florida | PacBio Sequel | - | GCF_014884745.1 | complete genome | 2 | 60 | 9 | 4717 | *R. solanacearum* | 3 |
| NCPPB 3985 | Peru | PacBio Sequel | *Solanum melongena* | GCF_015910635.1 | contig | 12 | 73 | 10 | 4780 | *R. solanacearum* | - |
| NCPPB 325* | USA | PacBio Sequel | *Solanum lycopersicum* | GCF_015910705.1 | contig | 19 | 66 | 14 | 4913 | *R. solanacearum* | - |
| IPO 0715 | Bangladesh | Illumina HiSeq | *Solanum tuberosum* | GCF_015910755.1 | contig | 170 | 61 | 3 | 4523 | *R. solanacearum* | - |
| PD 445 | Egypt | Illumina HiSeq | *Solanum tuberosum* | GCF_015910775.1 | contig | 150 | 61 | 3 | 4515 | *R. solanacearum* | - |
| IPO 0738 | - | Illumina HiSeq | *Solanum tuberosum* | GCF_015910785.1 | contig | 148 | 61 | 3 | 4513 | *R. solanacearum* | - |
| PD 141 | - | Illumina HiSeq | *Solanum tuberosum* | GCF_015910795.1 | contig | 186 | 61 | 3 | 4533 | *R. solanacearum* | - |
| PD 2762 | - | Illumina HiSeq | *Solanum tuberosum* | GCF_015910825.1 | contig | 267 | 61 | 4 | 4519 | *R. solanacearum* | - |
| PD 1447 | - | Illumina HiSeq | *Solanum tuberosum* | GCF_015910855.1 | contig | 137 | 62 | 2 | 5003 | *R. solanacearum* | - |
| PD 1414 | - | Illumina HiSeq | *Solanum tuberosum* | GCF_015910875.1 | contig | 369 | 62 | 3 | 4946 | *R. solanacearum* | - |
| PD 1446 | - | Illumina HiSeq | *Solanum tuberosum* | GCF_015910885.1 | contig | 115 | 62 | 3 | 4747 | *R. solanacearum* | - |
| PD 134 | - | Illumina HiSeq | *Solanum tuberosum* | GCF_015910925.1 | contig | 185 | 61 | 3 | 4515 | *R. solanacearum* | - |
| NAK 7 | - | Illumina HiSeq | *Solanum tuberosum* | GCF_015910965.1 | contig | 253 | 61 | 3 | 4533 | *R. solanacearum* | - |
| NAK 216 | - | Illumina HiSeq | - | GCF_015911015.1 | contig | 151 | 61 | 3 | 4517 | *R. solanacearum* | - |
| NAK 214 | - | Illumina HiSeq | - | GCF_015911025.1 | contig | 147 | 61 | 3 | 4515 | *R. solanacearum* | - |
| NAK 204 | - | Illumina HiSeq | - | GCF_015911055.1 | contig | 145 | 61 | 3 | 4514 | *R. solanacearum* | - |
| NAK 215 | - | Illumina HiSeq | - | GCF_015911065.1 | contig | 146 | 61 | 3 | 4514 | *R. solanacearum* | - |
| NAK 213 | - | Illumina HiSeq | - | GCF_015911075.1 | contig | 154 | 61 | 3 | 4522 | *R. solanacearum* | - |
| NAK 210 | - | Illumina HiSeq | - | GCF_015911095.1 | contig | 148 | 61 | 3 | 4521 | *R. solanacearum* | - |
| NAK 202 | - | Illumina HiSeq | - | GCF_015911115.1 | contig | 149 | 61 | 3 | 4519 | *R. solanacearum* | - |
| NAK 201 | - | Illumina HiSeq | - | GCF_015911155.1 | contig | 144 | 61 | 3 | 4513 | *R. solanacearum* | - |
| NAK 199 | - | Illumina HiSeq | - | GCF_015911165.1 | contig | 146 | 61 | 3 | 4516 | *R. solanacearum* | - |
| NAK 180 | - | Illumina HiSeq | - | GCF_015911195.1 | contig | 148 | 61 | 3 | 4526 | *R. solanacearum* | - |
| NAK 198 | - | Illumina HiSeq | - | GCF_015911205.1 | contig | 158 | 61 | 3 | 4522 | *R. solanacearum* | - |
| NAK 170 | - | Illumina HiSeq | - | GCF_015911215.1 | contig | 153 | 61 | 3 | 4537 | *R. solanacearum* | - |
| NAK 164 | - | Illumina HiSeq | - | GCF_015911255.1 | contig | 148 | 61 | 3 | 4522 | *R. solanacearum* | - |
| NAK 166 | - | Illumina HiSeq | - | GCF_015911275.1 | contig | 145 | 61 | 3 | 4509 | *R. solanacearum* | - |
| NAK 163 | - | Illumina HiSeq | - | GCF_015911295.1 | contig | 148 | 61 | 3 | 4525 | *R. solanacearum* | - |
| NAK 162 | - | Illumina HiSeq | - | GCF_015911305.1 | contig | 165 | 61 | 3 | 4515 | *R. solanacearum* | - |
| NAK 161 | - | Illumina HiSeq | - | GCF_015911355.1 | contig | 151 | 61 | 3 | 4528 | *R. solanacearum* | - |
| NAK 159 | - | Illumina HiSeq | - | GCF_015911395.1 | contig | 148 | 61 | 3 | 4513 | *R. solanacearum* | - |
| PD 4321 | Portugal | Illumina HiSeq | *Portulaca oleracea* | GCF_015911405.1 | contig | 280 | 61 | 3 | 4514 | *R. solanacearum* | - |
| PD 4124 | Kenya | Illumina HiSeq | *Pelargonium* sp. | GCF_015911415.1 | contig | 145 | 61 | 3 | 4507 | *R. solanacearum* | - |
| PD 3668 | Netherlands | Illumina HiSeq | *Solanum nigrum* | GCF_015911455.1 | contig | 395 | 60 | 3 | 4566 | *R. solanacearum* | - |
| PD 4027 | Netherlands | Illumina HiSeq | *Pelargonium* sp. | GCF_015911465.1 | contig | 354 | 61 | 3 | 4566 | *R. solanacearum* | - |
| PD 3270 | Costa Rica | Illumina HiSeq | *Solanum tuberosum* | GCF_015911515.1 | contig | 165 | 60 | 3 | 4834 | *R. solanacearum* | - |
| PD 3223 | Greece | Illumina HiSeq | *Solanum lycopersicum* | GCF_015911555.1 | contig | 191 | 61 | 3 | 4536 | *R. solanacearum* | - |
| PD 3269 | Slovenia | Illumina HiSeq | *Solanum tuberosum* | GCF_015911565.1 | contig | 166 | 61 | 3 | 4515 | *R. solanacearum* | - |
| PD:3222 | Ecuador | Illumina HiSeq | *Anthurium andraeanum* | GCF_015911585.1 | contig | 248 | 56 | 3 | 4894 | *R. solanacearum* | - |
| PD:3203 | Netherlands | Illumina HiSeq | *Solanum lycopersicum* | GCF_015911625.1 | contig | 223 | 61 | 3 | 4497 | *R. solanacearum* | - |
| PD:3121 | Netherlands | Illumina HiSeq | - | GCF_015911675.1 | contig | 419 | 60 | 3 | 4557 | *R. solanacearum* | - |
| PD:3115 | Netherlands | Illumina HiSeq | - | GCF_015911685.1 | contig | 385 | 60 | 3 | 4543 | *R. solanacearum* | - |
| PD:3049 | Egypt | Illumina HiSeq | - | GCF_015911715.1 | contig | 347 | 60 | 3 | 4559 | *R. solanacearum* | - |
| PD:2625 | Turkey | Illumina HiSeq | *Solanum tuberosum* | GCF_015911725.1 | contig | 498 | 61 | 3 | 4589 | *R. solanacearum* | - |
| IPO:3731 | Kenya | Illumina HiSeq | *Pelargonium* sp. | GCF_015911775.1 | contig | 194 | 61 | 3 | 4546 | *R. solanacearum* | - |
| PD:441 | Sweden | Illumina HiSeq | *Solanum tuberosum* | GCF_015911785.1 | contig | 131 | 61 | 3 | 4407 | *R. solanacearum* | - |
| IPO:1828 | - | Illumina HiSeq | *Solanum tuberosum* | GCF_015911795.1 | contig | 146 | 61 | 3 | 4512 | *R. solanacearum* | - |
| IPO:1808 | Netherlands | Illumina HiSeq | *Solanum tuberosum* | GCF_015911835.1 | contig | 149 | 61 | 3 | 4517 | *R. solanacearum* | - |
| IPO:1809 | Netherlands: Lemele, Overijssel | Illumina HiSeq | *-* | GCF_015911845.1 | contig | 199 | 61 | 3 | 4515 | *R. solanacearum* | - |
| IPO:1805 | Netherlands | Illumina HiSeq | *Solanum nigrum* | GCF_015911875.1 | contig | 148 | 61 | 3 | 4513 | *R. solanacearum* | - |
| IPO:1804 | Netherlands | Illumina HiSeq | - | GCF_015911895.1 | contig | 147 | 61 | 3 | 4511 | *R. solanacearum* | - |
| IPO:1806 | Netherlands | Illumina HiSeq | *Solanum nigrum* | GCF_015911905.1 | contig | 150 | 61 | 3 | 4511 | *R. solanacearum* | - |
| IPO:1752 |  | Illumina HiSeq | *Solanum melongena* | GCF_015911935.1 | contig | 159 | 61 | 3 | 4519 | *R. solanacearum* | - |
| CFBP:3581 | France | Illumina HiSeq | *Solanum lycopersicum* | GCF_015911945.1 | contig | 227 | 61 | 3 | 4501 | *R. solanacearum* | - |
| IPO:1750 | France | Illumina HiSeq | *Solanum lycopersicum* | GCF_015911975.1 | contig | 223 | 61 | 3 | 4531 | *R. solanacearum* | - |
| PD:2942 | Netherlands | Illumina HiSeq | *Solanum tuberosum* | GCF_015911985.1 | contig | 146 | 61 | 3 | 4513 | *R. solanacearum* | - |
| PD:2941 | Netherlands | Illumina HiSeq | *Solanum tuberosum* | GCF_015912005.1 | contig | 150 | 61 | 3 | 4510 | *R. solanacearum* | - |
| PD:2940 | Netherlands | Illumina HiSeq | *Solanum tuberosum* | GCF_015912035.1 | contig | 149 | 61 | 3 | 4513 | *R. solanacearum* | - |
| PD:1445 | Panama | Illumina HiSeq | *Musa* sp. | GCF_015912065.1 | contig | 108 | 60 | 3 | 4811 | *R. solanacearum* | - |
| PD:1260 | France | Illumina HiSeq Gj707 | *Solanum tuberosum* | GCF_015912085.1 | contig | 210 | 61 | 3 | 4523 | *R. solanacearum* | - |
| PD:1408 | Chile | Illumina HiSeq | *Solanum tuberosum* | GCF_015912105.1 | contig | 199 | 61 | 3 | 4561 | *R. solanacearum* | - |
| PD:1254 | India | Illumina HiSeq | *Solanum tuberosum* | GCF_015912135.1 | contig | 161 | 61 | 3 | 4526 | *R. solanacearum* | - |
| PD:2763 | Netherlands | PacBio Sequel | *Solanum tuberosum* | GCF_015912235.1 | contig | 36 | 61 | 3 | 4549 | *R. solanacearum* | - |
| GEO_57 | Georgia: Akhaltsikhe | Illumina MiSeq | *Solanum tuberosum* | GCA_002029885.1 | scaffold | 145 | 58 | 3 | 4497 | *R. solanacearum* | - |
| IPO1609 | Philippines:Mindanao | - | *Musa nana* | GCF_001050995.1 | scaffold | 10 | 40 | 4 | 4643 | *R. solanacearum* | - |
| GEO_99 | Georgia: Keda | Illumina MiSeq | *Capsicum annuum* | GCF_002029865.1 | scaffold | 147 | 54 | 3 | 4511 | *R. solanacearum* | - |
| GEO_96 | Georgia: Khelvachauri | Illumina MiSeq | *Solanum lycopersicum* | GCF_002029895.1 | scaffold | 129 | 58 | 3 | 4485 | *R. solanacearum* | - |
| GEO_6 | Georgia | Illumina | *Solanum tuberosum* | GCA_002894765.1 | contig | 123 | 58 | 3 | 4464 | *R. solanacearum* | - |
| P816 | USA: Florida | Illumina HiSeq | high bush blueberry | GCA_003337165.1 | scaffold | 142 | 48 | 9 | 3836 | *R. solanacearum* | - |
| 10314 | Philippines: Davao | IonTorrent | *Musa acuminata* | GCA_008271875.1 | contig | 391 | 58 | 3 | 4981 | *R. solanacearum* | - |
| CFBP 8697 | Iran | Illumina NovaSeq | *Solanum tuberosum* | GCA_009832785.1 | complete genome | 2 | 38 | 4 | 4272 | *R. solanacearum* | 1 |
| CFBP 8695 | Iran | Illumina NovaSeq | *Solanum tuberosum* | GCA_009832805.1 | complete genome | 2 | 39 | 4 | 4504 | *R. solanacearum* | 1 |
| CIAT_078 | Colombia | Oxford Nanopore MiniION | - | GCA_012562465.1 | complete genome | 2 | 61 | 7 | 4843 | *R. solanacearum* | 3 |
| UW72 | Greece | PacBio | - | GCA_021117095.1 | chromosome | 2 | 66 | 9 | 4595 | *R. solanacearum* | - |
| CIP417_UW70 | Colombia | PacBio | - | GCA_021117115.1 | chromosome | 2 | 61 | 9 | 4788 | *R. solanacearum* | - |
| UW251 | Colombia | PacBio | - | GCA_021117135.1 | chromosome | 2 | 66 | 9 | 4738 | *R. solanacearum* | - |
| RsT01 | Colombia | Illumina MiSeq | *Solanum lycopersicum* | GCA_022095055.1 | contig | 104 | 57 | 3 | 4657 | *R. solanacearum* | - |
| UW88 | Brazil | PacBio | - | GCA_023074875.1 | contig | 6 | 63 | 9 | 4903 | *R. solanacearum* | - |
| B73_UW840 | Brazil | Illumina | - | GCA_023074895.1 | contig | 151 | 60 | 3 | 4919 | *R. solanacearum* | - |
| B70_UW839 | Brazil | Illumina | - | GCA_023074915.1 | contig | 155 | 61 | 3 | 4917 | *R. solanacearum* | - |
| B68_UW837 | Brazil | Illumina | - | GCA_023074935.1 | contig | 174 | 60 | 3 | 4922 | *R. solanacearum* | - |
| UW82 | Colombia | PacBio | - | GCA_023074955.1 | contig | 4 | 65 | 9 | 4800 | *R. solanacearum* | - |
| UW87 | Canada | Illumina | - | GCA_023074995.1 | contig | 218 | 56 | 3 | 4751 | *R. solanacearum* | - |
| UW80 | Colombia | PacBio | - | GCA_023075045.1 | contig | 6 | 66 | 9 | 4873 | *R. solanacearum* | - |
| IBSBF2001_UW756 | Brazil | PacBio | - | GCA_023075135.1 | contig | 3 | 65 | 9 | 5090 | *R. solanacearum* | - |
| Rs540_UW751 | Brazil | Illumina | - | GCA_023075165.1 | contig | 186 | 60 | 3 | 4860 | *R. solanacearum* | - |
| Rs545_UW752 | Brazil | Illumina | - | GCA_023075175.1 | contig | 241 | 55 | 3 | 5072 | *R. solanacearum* | - |
| UW73 | Sri Lanka | PacBio | - | GCA_023075275.1 | contig | 3 | 66 | 9 | 4951 | *R. solanacearum* | - |
| AW1_UW701 | USA | Illumina | - | GCA_023075295.1 | contig | 246 | 58 | 3 | 4590 | *R. solanacearum* | - |
| CIP240_RUN482_UW692 | Brazil | PacBio | - | GCA_023075335.1 | contig | 9 | 63 | 9 | 5151 | *R. solanacearum* | - |
| T1-UY_UW688 | Uruguay | Illumina | - | GCA_023075355.1 | contig | 148 | 60 | 3 | 4926 | *R. solanacearum* | - |
| NCPPB3987_UW656 | Brazil | PacBio | - | GCA_023075395.1 | contig | 4 | 65 | 9 | 4726 | *R. solanacearum* | - |
| RUN58_UW646 | Reunion | Illumina | - | GCA_023075475.1 | contig | 85 | 62 | 3 | 4562 | *R. solanacearum* | - |
| CFBP7029_UW665 | Cameroon | Illumina | - | GCA_023075495.1 | contig | 135 | 61 | 3 | 4423 | *R. solanacearum* | - |
| CIP301_UW641 | Peru | Illumina | - | GCA_023075515.1 | contig | 83 | 60 | 3 | 4568 | *R. solanacearum* | - |
| CIP239_RUN043_UW639 | Brazil | Illumina | - | GCA_023075555.1 | contig | 196 | 56 | 3 | 4862 | *R. solanacearum* | - |
| UW599 | Guatemala | Illumina | - | GCA_023075655.1 | contig | 131 | 62 | 3 | 4350 | *R. solanacearum* | - |
| Rs124_UW586 | USA | Illumina | - | GCA_023075685.1 | contig | 232 | 59 | 3 | 4980 | *R. solanacearum* | - |
| UW592 | Guatemala | Illumina | - | GCA_023075695.1 | contig | 187 | 56 | 3 | 4424 | *R. solanacearum* | - |
| UW611 | Guatemala | Illumina | - | GCA_023075705.1 | contig | 168 | 62 | 3 | 4531 | *R. solanacearum* | - |
| UW573 | Guatemala | Illumina | - | GCA_023075715.1 | contig | 154 | 61 | 3 | 4465 | *R. solanacearum* | - |
| UW560 | Guatemala | Illumina | - | GCA_023075775.1 | contig | 143 | 62 | 3 | 4435 | *R. solanacearum* | - |
| UW552 | Guatemala | Illumina | - | GCA_023075785.1 | contig | 156 | 61 | 3 | 4427 | *R. solanacearum* | - |
| UW557 | Guatemala | Illumina | - | GCA_023075815.1 | contig | 158 | 61 | 3 | 4528 | *R. solanacearum* | - |
| IPO1609_UW550 | Netherlands | Illumina | - | GCA_023075825.1 | contig | 153 | 58 | 3 | 4475 | *R. solanacearum* | - |
| LNPV14-25_UW523 | France | Illumina | - | GCA_023075835.1 | contig | 142 | 61 | 3 | 4534 | *R. solanacearum* | - |
| CIP265_UW504 | Indonesia | Illumina | - | GCA_023075895.1 | contig | 139 | 61 | 3 | 4489 | *R. solanacearum* | - |
| CIP310_UW495 | Colombia | PacBio | - | GCA_023075915.1 | contig | 3 | 64 | 9 | 4724 | *R. solanacearum* | - |
| CIP180_UW500 | Indonesia | PacBio | - | GCA_023075955.1 | contig | 11 | 65 | 9 | 4779 | *R. solanacearum* | - |
| CIP309_UW494 | Colombia | Illumina | - | GCA_023075975.1 | contig | 141 | 59 | 3 | 4963 | *R. solanacearum* | - |
| CIP302_UW492 | Peru | Illumina | - | GCA_023075995.1 | contig | 203 | 59 | 3 | 4965 | *R. solanacearum* | - |
| CIP282_UW489 | Chile | Illumina | - | GCA_023076035.1 | contig | 135 | 60 | 3 | 4610 | *R. solanacearum* | - |
| CIP214_UW488 | Brazil | Illumina | - | GCA_023076055.1 | contig | 69 | 61 | 3 | 4671 | *R. solanacearum* | - |
| CIP167_UW484 | Peru | Illumina | - | GCA_023076075.1 | contig | 93 | 63 | 3 | 5019 | *R. solanacearum* | - |
| CIP177_UW485 | Peru | Illumina | - | GCA_023076095.1 | contig | 63 | 64 | 3 | 4698 | *R. solanacearum* | - |
| CIP207_UW487 | Peru | Illumina | - | GCA_023076105.1 | contig | 135 | 62 | 3 | 4578 | *R. solanacearum* | - |
| CIP9_UW476 | Costa Rica | Illumina | - | GCA_023076155.1 | contig | 99 | 62 | 3 | 4984 | *R. solanacearum* | - |
| CIP300_UW473 | Peru | Illumina | - | GCA_023076215.1 | contig | 156 | 58 | 3 | 4496 | *R. solanacearum* | - |
| CIP240_UW470 | Brazil | Illumina | - | GCA_023076235.1 | contig | 153 | 59 | 3 | 4967 | *R. solanacearum* | - |
| CIP239_UW469 | Brazil | Illumina | - | GCA_023076255.1 | contig | 223 | 55 | 3 | 4867 | *R. solanacearum* | - |
| CIP226_UW467 | Brazil | Illumina | - | GCA_023076265.1 | contig | 5 | 75 | 9 | 5032 | *R. solanacearum* | - |
| CIP217_UW462 | Brazil | PacBio | - | GCA_023076295.1 | contig | 5 | 65 | 9 | 4787 | *R. solanacearum* | - |
| CIP224_UW466 | Brazil | PacBio | - | GCA_023076315.1 | contig | 10 | 67 | 9 | 5252 | *R. solanacearum* | - |
| CIP211_UW461 | Brazil | PacBio | - | GCA_023076335.1 | contig | 6 | 66 | 9 | 4851 | *R. solanacearum* | - |
| CIP172_UW458 | Peru | PacBio | - | GCA_023076355.1 | contig | 3 | 68 | 9 | 4996 | *R. solanacearum* | - |
| CIP218_UW463 | Brazil | PacBio | - | GCA_023076395.1 | contig | 3 | 64 | 9 | 5023 | *R. solanacearum* | - |
| CIP221_UW464 | Brazil | PacBio | - | GCA_023076415.1 | contig | 6 | 65 | 9 | 4790 | *R. solanacearum* | - |
| CIP258_UW448 | Burundi | Illumina | - | GCA_023076435.1 | contig | 139 | 58 | 3 | 4523 | *R. solanacearum* | - |
| CIP61_UW451 | Peru | Illumina | - | GCA_023076455.1 | contig | 59 | 64 | 3 | 4655 | *R. solanacearum* | - |
| CIP77_UW452 | Peru | Illumina | - | GCA_023076475.1 | contig | 160 | 58 | 3 | 4785 | *R. solanacearum* | - |
| CIP259_UW449 | - | Illumina | - | GCA_023076485.1 | contig | 151 | 61 | 3 | 4534 | *R. solanacearum* | - |
| O1059_UW442 | Australia | Illumina | - | GCA_023076515.1 | contig | 142 | 61 | 3 | 4503 | *R. solanacearum* | - |
| 01020AS_UW437 | Australia | Illumina | - | GCA_023076535.1 | contig | 124 | 61 | 3 | 4349 | *R. solanacearum* | - |
| O223A_UW420 | Australia | Illumina | - | GCA_023076545.1 | contig | 135 | 58 | 3 | 4372 | *R. solanacearum* | - |
| O249_UW425 | Australia | Illumina | - | GCA_023076565.1 | contig | 164 | 62 | 3 | 4500 | *R. solanacearum* | - |
| O15A_UW408 | Australia | Illumina | - | GCA_023076575.1 | contig | 156 | 62 | 3 | 4509 | *R. solanacearum* | - |
| UW37 | Colombia | PacBio | - | GCA_023076655.1 | contig | 5 | 66 | 9 | 4728 | *R. solanacearum* | - |
| 40-4SP_UW354 | Brazil | PacBio | - | GCA_023076695.1 | contig | 3 | 65 | 9 | 4632 | *R. solanacearum* | - |
| 26-3SP_UW351 | Brazil | PacBio | - | GCA_023076735.1 | contig | 4 | 65 | 9 | 4948 | *R. solanacearum* | - |
| 23-10BR_UW349 | Brazil | PacBio | - | GCA_023076755.1 | contig | 3 | 68 | 9 | 4962 | *R. solanacearum* | - |
| 25-2SP_UW350 | Brazil | Illumina | - | GCA_023076775.1 | contig | 130 | 57 | 3 | 4917 | *R. solanacearum* | - |
| 19-3PR_UW348 | Brazil | PacBio | - | GCA_023076795.1 | contig | 5 | 67 | 9 | 5015 | *R. solanacearum* | - |
| 17-4SC_UW347 | Brazil | PacBio | - | GCA_023076805.1 | contig | 7 | 67 | 9 | 4826 | *R. solanacearum* | - |
| 15-3SC_UW346 | Brazil | PacBio | - | GCA_023076835.1 | contig | 5 | 65 | 9 | 5224 | *R. solanacearum* | - |
| 14-2SC_UW345 | Brazil | PacBio | - | GCA_023076855.1 | contig | 7 | 64 | 9 | 5069 | *R. solanacearum* | - |
| 10-1SC_UW344 | Brazil | PacBio | - | GCA_023076865.1 | contig | 6 | 66 | 9 | 4805 | *R. solanacearum* | - |
| UW330 | Guyana | Illumina | - | GCA_023076905.1 | contig | 149 | 58 | 3 | 4310 | *R. solanacearum* | - |
| UW28 | Cyprus | PacBio | - | GCA_023076945.1 | contig | 5 | 67 | 9 | 5229 | *R. solanacearum* | - |
| CIP90_UW274 | Costa Rica | Illumina | - | GCA_023076975.1 | contig | 148 | 61 | 3 | 4525 | *R. solanacearum* | - |
| UW276 | Mexico | Illumina | - | GCA_023077005.1 | contig | 157 | 60 | 3 | 4531 | *R. solanacearum* | - |
| UW273 | Costa Rica | PacBio | - | GCA_023077035.1 | contig | 4 | 64 | 9 | 4740 | *R. solanacearum* | - |
| UW272 | Costa Rica | Illumina | - | GCA_023077055.1 | contig | 115 | 61 | 3 | 4957 | *R. solanacearum* | - |
| UW263 | Peru | PacBio | - | GCA_023077075.1 | contig | 3 | 66 | 9 | 4871 | *R. solanacearum* | - |
| UW262 | Peru | PacBio | - | GCA_023077095.1 | contig | 4 | 66 | 9 | 4551 | *R. solanacearum* | - |
| UW261 | Peru | Illumina | - | GCA_023077115.1 | contig | 62 | 60 | 3 | 4649 | *R. solanacearum* | - |
| UW257 | Costa Rica | Illumina | - | GCA_023077155.1 | contig | 145 | 61 | 3 | 4518 | *R. solanacearum* | - |
| UW256 | Costa Rica | Illumina | - | GCA_023077175.1 | contig | 125 | 60 | 3 | 4822 | *R. solanacearum* | - |
| UW24 | Israel | Illumina | - | GCA_023077195.1 | contig | 124 | 61 | 3 | 4451 | *R. solanacearum* | - |
| UW260 | Peru | PacBio | - | GCA_023077215.1 | contig | 4 | 66 | 9 | 4696 | *R. solanacearum* | - |
| UW23 | Israel | Illumina | - | GCA_023077235.1 | contig | 173 | 61 | 3 | 4568 | *R. solanacearum* | - |
| UW225 | - | Illumina | - | GCA_023077255.1 | contig | 157 | 63 | 3 | 4356 | *R. solanacearum* | - |
| UW229 | Brazil | PacBio | - | GCA_023077275.1 | contig | 2 | 65 | 9 | 4653 | *R. solanacearum* | - |
| UW224 | Kenya | Illumina | - | GCA_023077295.1 | contig | 148 | 58 | 3 | 4373 | *R. solanacearum* | - |
| UW220e | India | Illumina | - | GCA_023077315.1 | contig | 139 | 62 | 3 | 4508 | *R. solanacearum* | - |
| UW220c | India | Illumina | - | GCA_023077335.1 | contig | 163 | 61 | 3 | 4502 | *R. solanacearum* | - |
| UW220b | India | Illumina | - | GCA_023077355.1 | contig | 153 | 61 | 3 | 4534 | *R. solanacearum* | - |
| UW210 | USA | Illumina | - | GCA_023077375.1 | contig | 195 | 57 | 3 | 4787 | *R. solanacearum* | - |
| UW220a | India | Illumina | - | GCA_023077415.1 | contig | 148 | 58 | 3 | 4503 | *R. solanacearum* | - |
| UW187 | USA | Illumina | - | GCA_023077435.1 | contig | 182 | 56 | 3 | 4751 | *R. solanacearum* | - |
| UW175 | Colombia | Illumina | - | GCA_023077475.1 | contig | 109 | 57 | 3 | 4687 | *R. solanacearum* | - |
| UW162 | Peru | PacBio | - | GCA_023077495.1 | contig | 10 | 61 | 9 | 5024 | *R. solanacearum* | - |
| UW161 | Peru | PacBio | - | GCA_023077515.1 | contig | 4 | 61 | 9 | 4949 | *R. solanacearum* | - |
| UW160 | Peru | PacBio | - | GCA_023077535.1 | contig | 11 | 61 | 9 | 5074 | *R. solanacearum* | - |
| UW153 | Australia | Illumina | - | GCA_023077555.1 | contig | 190 | 58 | 3 | 4654 | *R. solanacearum* | - |
| UW156 | Peru | PacBio | - | GCA_023077575.1 | contig | 5 | 62 | 9 | 4939 | *R. solanacearum* | - |
| UW145 | Australia | PacBio | - | GCA_023077585.1 | contig | 102 | 57 | 3 | 4761 | *R. solanacearum* | - |
| UW134 | Kenya | PacBio | - | GCA_023077615.1 | contig | 3 | 61 | 9 | 4987 | *R. solanacearum* | - |
| UW120 | Costa Rica | Illumina | - | GCA_023077635.1 | contig | 152 | 58 | 3 | 4515 | *R. solanacearum* | - |
| OR214 | - | Illumina MiSeq; Roche 454 | - | GCA_000372665.1 | contig | - | - | - | - | Unclassified *Ralstonia* spp. | - |
| JGI 0001001-A05 | - | Illumina HiSeq 2000 | - | GCA_000485415.1 | scaffold | - | - | - | - | Unclassified *Ralstonia* spp. | - |
| SSH4 | USA: California | 454 | - | GCA_000607165.1 | scaffold | - | - | - | - | Unclassified *Ralstonia* spp. | - |
| CW2 | - | 454 | - | GCA_000607185.1 | contig | - | - | - | - | Unclassified *Ralstonia* spp. | - |
| UNC404CL21Col | - | Illumina HiSeq 2000 | - | GCA_000620465.1 | scaffold | - | - | - | - | Unclassified *Ralstonia* spp. | - |
| A12 | Malaysia: Rimba Ilmu | Illumina MiSeq | - | GCA_000801955.1 | contig | - | - | - | - | Unclassified *Ralstonia* spp. | - |
| H2Cu5 | Portugal | IonTorrent | - | GCA_001699815.1 | contig | - | - | - | - | Unclassified *Ralstonia* spp. | - |
| FJAT-462 | China: Fujian | Illumina HiSeq | *Capsicum annuum* | GCA_001920895.2 | scaffold | - | - | - | - | Unclassified *Ralstonia* spp. | - |
| FJAT-452 | China: Fujian | Illumina HiSeq | *Solanum melongena* | GCA_001920905.2 | scaffold | - | - | - | - | Unclassified *Ralstonia* spp. | - |
| UBA689 | NULL | Illumina | - | GCA_002298975.1 | scaffold | - | - | - | - | Unclassified *Ralstonia* spp. | - |
| 52 | China: Zhejiang Province | Illumina HiSeq | - | GCA_002849525.1 | contig | - | - | - | - | Unclassified *Ralstonia* spp. | - |
| GX3-BWBA | China: Guangxi | Illumina HiSeq | - | GCA_003290055.1 | contig | - | - | - | - | Unclassified *Ralstonia* spp. | - |
| SET104 | Japan:Hyogo | Illumina NEXTSeq 500 | - | GCA_003851545.1 | contig | - | - | - | - | Unclassified *Ralstonia* spp. | - |
| 3PA37C10 | Italy: Trento | Illumina MiSeq | - | GCA_005503495.1 | contig | - | - | - | - | Unclassified *Ralstonia* spp. | - |
| TCR112 | Japan: Gifu City, Gifu Prefecture | Illumina MiSeq; IonTorrent | *Allium tuberosum* | GCA_007997035.1 | contig | - | - | - | - | Unclassified *Ralstonia* spp. | - |
| s27 | USA: International Space Station | Illumina NextSeq | - | GCA_014142205.1 | scaffold | - | - | - | - | Unclassified *Ralstonia* spp. | - |
| s26 | USA: International Space Station | Illumina NextSeq | - | GCA_014142265.1 | contig | - | - | - | - | Unclassified *Ralstonia* spp. | - |
| s17 | USA: International Space Station | Illumina NextSeq | - | GCA_014142445.1 | contig | - | - | - | - | Unclassified *Ralstonia* spp. | - |
| s14 | USA: International Space Station | Illumina NextSeq | - | GCA_014142505.1 | contig | - | - | - | - | Unclassified *Ralstonia* spp. | - |
| s13 | USA: International Space Station | Illumina NextSeq | - | GCA_014142515.1 | contig | - | - | - | - | Unclassified *Ralstonia* spp. | - |
| s11 | USA: International Space Station | Illumina NextSeq | - | GCA_014142545.1 | contig | - | - | - | - | Unclassified *Ralstonia* spp. | - |
| s10 | USA: International Space Station | Illumina NextSeq | - | GCA_014142575.1 | scaffold | - | - | - | - | Unclassified *Ralstonia* spp. | - |
| s1 | USA: International Space Station | Illumina NextSeq | - | GCA_014142725.1 | scaffold | - | - | - | - | Unclassified *Ralstonia* spp. | - |
| s2 | USA: International Space Station | Illumina NextSeq | - | GCA_014142755.1 | contig | - | - | - | - | Unclassified *Ralstonia* spp. | - |
| ASV6 | USA: Massachusetts | Illumina NovaSeq | *Sarracenia purpurea* | GCA_016429285.1 | contig | - | - | - | - | Unclassified *Ralstonia* spp. | - |
| NCTR106 | USA | Illumina MiSeq | - | GCA_018603815.1 | contig | - | - | - | - | Unclassified *Ralstonia* spp. | - |
| 110760020-1 | - | Illumina NextSeq 500 | - | GCA_019642915.1 | contig | - | - | - | - | Unclassified *Ralstonia* spp. | - |
| 110700041-2 | - | Illumina NextSeq 500 | - | GCA_019642935.1 | contig | - | - | - | - | Unclassified *Ralstonia* spp. | - |
| 09216007 FQY_4 | - | Illumina NextSeq 500 | - | GCA_019642995.1 | contig | - | - | - | - | Unclassified *Ralstonia* spp. | - |
| 102690001-2 | - | Illumina NextSeq 500 | - | GCA_019643105.1 | contig | - | - | - | - | Unclassified *Ralstonia* spp. | - |
| 101480038-1 | - | Illumina NextSeq 500 | - | GCA_019643135.1 | contig | - | - | - | - | Unclassified *Ralstonia* spp. | - |
| 101120021-1 | - | Illumina NextSeq 500 | - | GCA_019643195.1 | contig | - | - | - | - | Unclassified *Ralstonia* spp. | - |
| 101480035-1 | - | Illumina NextSeq 500 | - | GCA_019643215.1 | contig | - | - | - | - | Unclassified *Ralstonia* spp. | - |
| 100540015-2 | - | Illumina NextSeq 500 | - | GCA_019643235.1 | contig | - | - | - | - | Unclassified *Ralstonia* spp. | - |
| 15-1563-3 | - | Oxford Nanopore MinION; Illumina NovaSeq | - | GCA_022631215.1 | complete genome | - | - | - | - | Unclassified *Ralstonia* spp. | - |
| ACH732_UW629 | Australia | Illumina | - | GCA_023075575.1 | contig | - | - | - | - | Unclassified *Ralstonia* spp. | - |
| TS | United Kingdom: University of Surrey | Illumina HiSeq | - | GCA_023217975.1 | contig | - | - | - | - | Unclassified *Ralstonia* spp. | - |
| 25MFCol4.1 | - | - | - | GCA_900104095.1 | scaffold | - | - | - | - | Unclassified *Ralstonia* spp. | - |
| NFACC01 | - | - | - | GCA_900115545.1 | scaffold | - | - | - | - | Unclassified *Ralstonia* spp. | - |
| bin465 | China | Illumina HiSeq | - | GCA_020350765.1 | chromosome | - | - | - | - | Unclassified *Ralstonia* spp. | - |

Table S2. Average nucleotide identity values between strains of “unclassified *Ralstonia* spp.” group and type strains of closely related *Ralstonia* species

| Subgroup | Assembly ID | FastANI (%) | | | | | |
| --- | --- | --- | --- | --- | --- | --- | --- |
|  |  | *R. insidiosa* | *R. mannitolilytica* | *R. pickettii* | *R. pseudosolanacearum* | *R. solanacearum* | *R. syzygii* |
| RSSC-like | GCA_001920895.2 | 83.23 | 83.98 | 83.65 | 94.51 | 90.07 | 90.87 |
|  | GCA_001920905.2 | 83.34 | 84.07 | 83.65 | 94.67 | 90.39 | 91.13 |
|  | GCA_023075575.1 | 83.66 | 84.68 | 83.60 | 91.97 | 91.18 | 93.50 |
| Others | GCA_003851545.1 | 86.78 | 86.41 | 88.21 | 83.90 | 83.71 | 84.06 |
|  | GCA_002298975.1 | 86.76 | 86.28 | 86.07 | 84.88 | 84.51 | 84.93 |
|  | GCA_900104095.1 | 78.11 | 78.81 | 78.10 | 79.16 | 78.74 | 79.04 |
|  | GCA_020350765.1 | 85.57 | 85.39 | 84.86 | 85.57 | 85.24 | 85.38 |
| Ri/Rpi-like | GCA_000372665.1 | 86.72 | 87.52 | 94.97 | 83.78 | 83.36 | 83.99 |
|  | GCA_000485415.1 | 86.56 | 87.66 | 94.83 | 83.70 | 83.32 | 83.85 |
|  | GCA_000607165.1 | 86.21 | 86.98 | 91.17 | 83.48 | 83.14 | 83.71 |
|  | GCA_000607185.1 | 86.22 | 87.57 | 94.86 | 83.83 | 83.26 | 83.91 |
|  | GCA_000620465.1 | 86.94 | 88.58 | 92.78 | 84.09 | 83.86 | 84.17 |
|  | GCA_000801955.1 | 90.04 | 86.19 | 86.50 | 84.20 | 83.83 | 84.49 |
|  | GCA_001699815.1 | 86.68 | 87.73 | 94.99 | 83.81 | 83.53 | 83.83 |
|  | GCA_002849525.1 | 86.84 | 88.52 | 92.46 | 84.08 | 83.74 | 84.23 |
|  | GCA_003290055.1 | 86.05 | 86.84 | 91.19 | 83.49 | 83.08 | 83.68 |
|  | GCA_005503495.1 | 86.87 | 87.60 | 94.97 | 83.79 | 83.40 | 83.99 |
|  | GCA_007997035.1 | 86.64 | 88.08 | 92.01 | 84.00 | 83.74 | 84.12 |
|  | GCA_014142205.1 | 86.34 | 87.62 | 94.79 | 83.63 | 83.29 | 83.81 |
|  | GCA_014142265.1 | 86.34 | 87.62 | 94.81 | 83.66 | 83.34 | 83.87 |
|  | GCA_014142445.1 | 86.35 | 87.65 | 94.88 | 83.71 | 83.40 | 83.95 |
|  | GCA_014142505.1 | 86.34 | 87.57 | 94.82 | 83.60 | 83.34 | 83.83 |
|  | GCA_014142515.1 | 86.33 | 87.63 | 94.82 | 83.69 | 83.34 | 83.85 |
|  | GCA_014142545.1 | 86.33 | 87.62 | 94.82 | 83.64 | 83.29 | 83.85 |
|  | GCA_014142575.1 | 86.36 | 87.68 | 94.88 | 83.76 | 83.38 | 83.94 |
|  | GCA_014142725.1 | 86.32 | 87.58 | 94.80 | 83.58 | 83.28 | 83.81 |
|  | GCA_014142755.1 | 86.27 | 87.61 | 94.81 | 83.64 | 83.26 | 83.80 |
|  | GCA_016429285.1 | 91.34 | 85.84 | 86.01 | 83.77 | 83.38 | 84.04 |
|  | GCA_018603815.1 | 86.81 | 87.30 | 94.95 | 83.84 | 83.45 | 84.09 |
|  | GCA_019642915.1 | 86.35 | 87.62 | 94.80 | 83.63 | 83.29 | 83.83 |
|  | GCA_019642935.1 | 86.34 | 87.62 | 94.81 | 83.66 | 83.34 | 83.87 |
|  | GCA_019642995.1 | 86.27 | 87.61 | 94.81 | 83.64 | 83.26 | 83.80 |
|  | GCA_019643105.1 | 86.35 | 87.65 | 94.88 | 83.71 | 83.40 | 83.95 |
|  | GCA_019643135.1 | 86.34 | 87.57 | 94.82 | 83.60 | 83.34 | 83.83 |
|  | GCA_019643195.1 | 86.33 | 87.62 | 94.82 | 83.64 | 83.29 | 83.85 |
|  | GCA_019643215.1 | 86.33 | 87.63 | 94.82 | 83.69 | 83.34 | 83.85 |
|  | GCA_019643235.1 | 86.35 | 87.68 | 94.86 | 83.77 | 83.37 | 83.93 |
|  | GCA_022631215.1 | 93.77 | 86.53 | 86.69 | 84.00 | 83.62 | 84.26 |
|  | GCA_023217975.1 | 93.76 | 86.45 | 86.52 | 83.96 | 83.56 | 84.24 |
|  | GCA_900115545.1 | 86.55 | 87.31 | 91.45 | 83.62 | 83.42 | 83.74 |

The 40 strains in “unclassified *Ralstonia* spp.” group were divided into 3 subgroups according to ANI value. A total of 33 strains belonged to the “Ri/Rpi-like” subgroup, which had highest ANI values to type strain of *R. insidiosa* or *R. picketii* (90.04-94.99%) and lower than 85% ANI values to type strains of RSSC species. The “RSSC-like” subgroup contained only 3 strains (FJAT-462, FJAT-452 and ACH732_UW629), which had highest ANI values when compared with LMG 9673 and LMG 10661 (94.51% to LMG 9673, 94.67% to LMG 9673 and 93.50% to LMG 10661 respectively) and much lower ANI values (less than 85%) to type strains of other non-RSSC species. The remaining 4 strains were grouped in “Others” subgroup, which had ANI values lower than 90% to all type strains.

Table S3. Specificity of *Ralstonia* spp. and limit of detection of *R. pickettii* JCM 5969 and *R. pseudosolanacearum* LMG 9673

| Species | Strain | Consentration of genome DNA | TaqMan-based qPCR Ct value^a^ |
| --- | --- | --- | --- |
| *R. insidiosa* | LMG 21421 | 1 ng/μL | -^b^ |
| *R. pickettii* | JCM 5969 | 1 ng/μL | - |
| *R. mannitolilytica* | LMG 6866 | 1 ng/μL | 29.09^c^ |
|  |  | 0.1 ng/μL | 32.17 |
|  |  | 10 pg/μL | 35.31 |
|  |  | 1 pg/μL | - |
|  |  | 0.1 pg/μL | - |
|  |  | 10 fg/μL | - |
| *R. pseudosolanacearum* | LMG 9673 | 1 ng/μL | 20.60 |
|  |  | 0.1 ng/μL | 24.05 |
|  |  | 10 pg/μL | 28.37 |
|  |  | 1 pg/μL | 31.72 |
|  |  | 0.1 pg/μL | 34.46 |
|  |  | 10 fg/μL | 36.29 |

^a^: Detected by primers RSSC-F/Rs16S-R and TaqMan probe RSSC-P.

^b^: No signal was detected.

^c^: Average Ct value of three biological replicates.

Table S4. Estimation of the REs of *R. solanacarum* strain NCPPB 325 and *R. syzygii* LLRS-1 in three representative soils

| Strains name | Locations | Soil type^a^ | Target strain | |  | RsPC | | Corrected RE of target strain (%) | Relative accuracy before normalization | Relative accuracy after normalization |
| --- | --- | --- | --- | --- | --- | --- | --- | --- | --- | --- |
|  |  |  | RE (%)^b^ | Std (%)^c^ |  | RE (%) | Std (%) |  |  |  |
| NCPPB 325 | Kunming | heavy clay | 29.18 | 6.64 |  | 56.35 | 17.78 | 51.78 | 1.78 | 0.95 |
|  | Chengdu | silty loam | 54.40 | 7.83 |  | 51.34 | 8.29 | 105.96 | 0.88 | -0.08 |
|  | Wulanchabu | sandy loam | 45.69 | 14.39 |  | 62.24 | 2.71 | 73.41 | 1.13 | 0.45 |
|  |  |  |  |  |  |  |  |  |  |  |
| LLRS-1 | Kunming | heavy clay | 0.17 | 0.08 |  | 0.22 | 0.08 | 79.53 | 9.18 | 0.33 |
|  | Chengdu | silty loam | 77.60 | 7.88 |  | 69.82 | 4.34 | 111.15 | 0.37 | -0.15 |
|  | Wulanchabu | sandy loam | 52.97 | 4.01 |  | 52.02 | 5.91 | 101.83 | 0.92 | -0.03 |

^a^: soil textures were determined according to the international system of soil texture classification;

^b^: recovery efficiency;

^c^: standard deviation;

Target strain NCPPB 325 (3.03×10^4^ CFU) was spiked together with RsPC (2.20×10^4^ CFU) with three biological replicates. Target strain LLRS-1 (5.33×10^5^ CFU) was spiked together with RsPC (8.32×10^5^ CFU) with three biological replicates. DNA extraction of *R. solanacearum* NCPPB 325 and its RsPC was performed using DNeasy PowerSoil Kit and DNeasy PowerSoil Pro Kit was used for *R. syzygii* LLRS-1 and its RsPC. The relative accuracy = Log_2_(1/RE). REs and standard deviations were calculated based on three biological replicates. QPCR detections for each biological replicate were performed in four technical replicates.

Table S5. Information of soil samples used in the study

| Soil sample | pH | OM^a^ | Clay (%) | Silt (%) | Sand (%) | Longitude (°) | Latitude (°) | Crop | Time |
| --- | --- | --- | --- | --- | --- | --- | --- | --- | --- |
| Wulanchabu | 8.17 | 1.19 | 2.760 | 12.460 | 84.780 | 114.204563 | 41.946963 | potao | 2015 |
| Shanghai | 5.37 | 2.98 | 2.846 | 32.947 | 64.207 | - | - | Chinese cabbage | 2016 |
| Xingtai | 7.37 | 2.24 | 3.328 | 36.587 | 60.085 | 115.962067 | 37.841767 | cotton | 2015 |
| Jiayuguan | 8.08 | 1.85 | 4.268 | 40.653 | 55.079 | 98.343261 | 39.781600 | sunflower | 2017 |
| Bayannaoer | 7.58 | 2.16 | 4.971 | 42.417 | 52.612 | 108.189540 | 41.052670 | sunflower | 2015 |
| Haerbin | 6.83 | 3.56 | 2.065 | 54.846 | 43.089 | - | - | eggplant | 2017 |
| Shenyang | 6.26 | 2.45 | 2.082 | 49.850 | 48.068 | - | - | Chinese cabbage | 2011 |
| Chengdu | 5.33 | 4.10 | 3.255 | 65.707 | 31.038 | - | - | Chinese cabbage | 2013 |
| Nanchang | 5.76 | 5.35 | 5.937 | 54.713 | 39.350 | - | - | Chinese cabbage | 2013 |
| Kunming | 5.46 | 3.13 | 65.16 | 23.990 | 10.849 | 103.084722 | 25.959166 | potato | 2017 |

^a^: organic matter

Table S6. Ct values of detection of LMG 9673 and RsPC in ten different soil samples

| soil sample | biological replicate | RSSC qPCR assay | | | ISPC qPCR assay | | |
| --- | --- | --- | --- | --- | --- | --- | --- |
| Bayannaoer | 1 | 28.344 | 28.394 | 28.458 | 31.504 | 31.231 | 30.800 |
|  | 2 | 28.564 | 28.423 | 28.555 | 31.205 | 31.034 | 30.730 |
|  | 3 | 28.359 | 28.223 | 28.203 | 30.835 | 30.543 | 30.875 |
| Chengdu | 1 | 28.184 | 27.793 | 27.909 | 30.052 | 30.058 | 29.800 |
|  | 2 | 28.086 | 27.745 | 27.732 | 29.956 | 30.009 | 30.025 |
|  | 3 | 28.143 | 27.957 | 27.889 | 30.340 | 30.301 | 30.171 |
| Xingtai | 1 | 27.839 | 27.863 | 28.053 | 30.972 | 30.695 | 31.035 |
|  | 2 | 28.377 | 28.041 | 28.421 | 31.303 | 31.262 | 30.839 |
|  | 3 | 27.702 | 27.686 | 27.802 | 29.363 | 29.096 | 29.414 |
| Haerbin | 1 | 28.713 | 28.794 | 28.844 | 31.326 | 31.290 | 31.187 |
|  | 2 | 28.804 | 28.713 | 28.972 | 31.658 | 31.717 | 31.730 |
|  | 3 | 28.070 | 28.024 | 28.168 | 30.932 | 30.959 | 31.085 |
| Kunming | 1 | 29.487 | 29.532 | 29.565 | 32.090 | 32.007 | 31.373 |
|  | 2 | 30.216 | 30.188 | 30.106 | 31.194 | 31.044 | 31.406 |
|  | 3 | 29.529 | 29.214 | 29.160 | 31.304 | 31.511 | 31.549 |
| Wulanchabu | 1 | 28.249 | 27.997 | 28.167 | 30.393 | 30.342 | 30.168 |
|  | 2 | 28.011 | 27.778 | 27.780 | 30.080 | 30.105 | 29.615 |
|  | 3 | 27.947 | 27.713 | 27.641 | 29.902 | 29.839 | 29.920 |
| Jiayuguan | 1 | 27.986 | 27.740 | 27.884 | 30.598 | 30.722 | 30.792 |
|  | 2 | 27.907 | 27.960 | 28.008 | 31.023 | 31.228 | 30.523 |
|  | 3 | 28.200 | 27.689 | 28.042 | 30.713 | 30.694 | 31.268 |
| Nanchang | 1 | 27.779 | 27.753 | 27.822 | 30.333 | 30.222 | 30.102 |
|  | 2 | 28.035 | 27.954 | 28.093 | 30.663 | 30.585 | 30.950 |
|  | 3 | 27.845 | 27.898 | 27.923 | 30.480 | 30.372 | 30.774 |
| Shanghai | 1 | 27.592 | 27.383 | 27.369 | 29.757 | 29.799 | 29.803 |
|  | 2 | 28.117 | 27.986 | 28.143 | 30.619 | 30.631 | 30.327 |
|  | 3 | 27.678 | 27.556 | 27.574 | 29.957 | 30.293 | 30.150 |
| Shenyang | 1 | 28.332 | 28.223 | 28.277 | 30.869 | 30.841 | 30.861 |
|  | 2 | 27.627 | 27.542 | 27.601 | 30.155 | 30.167 | 30.142 |
|  | 3 | 28.160 | 28.043 | 28.015 | 29.970 | 29.959 | 30.158 |

Table S7. Ct values of detection of RsPC and LMG 9673 at different concentrations in three representative soil samples

| Soil sample | Population of target (CFU) | Biological replicate | RSSC qPCR assay | | | ISPC qPCR assay | | |
| --- | --- | --- | --- | --- | --- | --- | --- | --- |
| Wulanchabu | 1.55×10^5^ | 1 | 25.274 | 25.214 | 25.266 | 30.584 | 31.112 | 30.502 |
|  |  | 2 | 25.285 | 25.278 | 24.963 | 30.542 | 30.703 | 29.875 |
|  |  | 3 | 24.871 | 24.916 | 24.893 | 30.100 | 30.221 | 30.387 |
|  | 1.55×10^4^ | 1 | 28.253 | 28.236 | 28.344 | 29.870 | 29.648 | 29.836 |
|  |  | 2 | 28.634 | 28.689 | 28.648 | 29.751 | 29.885 | 30.103 |
|  |  | 3 | 28.707 | 28.798 | 28.793 | 30.539 | 30.620 | 30.457 |
|  | 1.55×10^3^ | 1 | 32.635 | 31.832 | 32.286 | 29.999 | 29.846 | 30.014 |
|  |  | 2 | 32.155 | 32.781 | 32.692 | 30.393 | 30.376 | 30.200 |
|  |  | 3 | 32.357 | 32.067 | 32.845 | 30.219 | 29.997 | 30.607 |
| Chengdu | 1.55×10^5^ | 1 | 24.892 | 24.888 | 24.876 | 30.225 | 30.342 | 30.201 |
|  |  | 2 | 25.228 | 24.878 | 24.855 | 30.053 | 29.597 | 30.203 |
|  |  | 3 | 24.968 | 24.967 | 24.946 | 30.186 | 30.135 | 30.216 |
|  | 1.55×10^4^ | 1 | 28.033 | 28.103 | 28.003 | 29.847 | 29.968 | 30.096 |
|  |  | 2 | 28.802 | 28.776 | 28.820 | 30.829 | 31.036 | 30.742 |
|  |  | 3 | 28.746 | 28.750 | 28.752 | 30.599 | 30.695 | 30.648 |
|  | 1.55×10^3^ | 1 | 32.202 | 32.045 | 31.918 | 30.717 | 30.549 | 30.374 |
|  |  | 2 | 31.587 | 31.910 | 31.502 | 30.000 | 30.297 | 30.231 |
|  |  | 3 | 32.294 | 32.133 | 32.154 | 30.685 | 30.515 | 30.177 |
| Kunming | 1.55×10^5^ | 1 | 27.222 | 26.787 | 26.829 | 31.060 | 31.651 | 31.814 |
|  |  | 2 | 26.848 | 27.181 | 26.884 | 31.832 | 33.152 | 31.408 |
|  |  | 3 | 25.882 | 26.181 | 26.157 | 31.423 | 31.358 | 31.054 |
|  | 1.55×10^4^ | 1 | 30.011 | 30.014 | 29.857 | 31.461 | 31.186 | 31.381 |
|  |  | 2 | 29.190 | 29.534 | 29.099 | 31.209 | 31.386 | 30.920 |
|  |  | 3 | 30.063 | 29.795 | 29.900 | 31.845 | 31.628 | 31.143 |
|  | 1.55×10^3^ | 1 | 32.383 | 32.109 | 32.008 | 31.270 | 31.323 | 31.313 |
|  |  | 2 | 34.973 | 33.557 | 34.602 | 32.268 | 31.871 | 31.343 |
|  |  | 3 | 31.216 | 31.466 | 31.381 | 31.372 | 31.434 | 31.221 |

Table S8. Ct values of detection of NCPPB 325 and LMG 10661 in three representative soil samples

| Strain | Soil sample | Biological replicate | RSSC qPCR assay | | | | ISPC qPCR assay | | | |
| --- | --- | --- | --- | --- | --- | --- | --- | --- | --- | --- |
| NCPPB 325 | Chengdu | 1 | 29.02 | 28.51 | 28.68 | 29.49 | 31.51 | 30.46 | 31.03 | 31.79 |
|  |  | 2 | 28.30 | 28.43 | 28.70 | 28.56 | 31.41 | 30.85 | 31.07 | 30.80 |
|  |  | 3 | 28.92 | 28.88 | 28.65 | 28.40 | 31.79 | 31.15 | 31.54 | 31.67 |
|  | Kunming | 1 | 30.04 | 28.83 | 30.05 | 29.60 | 31.56 | 31.04 | 31.13 | 31.89 |
|  |  | 2 | 29.41 | 29.36 | 30.73 | 30.51 | 31.43 | 31.59 | 31.51 | 31.12 |
|  |  | 3 | 29.28 | 29.52 | 28.63 | 29.84 | 30.33 | 30.34 | 31.13 | 30.67 |
|  | Wulanchabu | 1 | 29.36 | 28.91 | 28.99 | 29.53 | 30.90 | 30.53 | 31.32 | 31.33 |
|  |  | 2 | 29.37 | 29.95 | 28.85 | 29.09 | 31.15 | 30.65 | 30.92 | 30.99 |
|  |  | 3 | 28.18 | 28.41 | 28.66 | 28.76 | 31.01 | 31.10 | 31.21 | 30.23 |
| LLRS-1 | Wulanchabu | 1 | 25.00 | 24.85 | 23.80 | 23.86 | 25.37 | 25.97 | 25.90 | 25.65 |
|  |  | 2 | 25.03 | 24.53 | 24.62 | 23.88 | 25.12 | 25.53 | 25.47 | 25.54 |
|  |  | 3 | 24.88 | 24.46 | 24.40 | 24.66 | 25.36 | 25.57 | 25.81 | 26.05 |
|  | Chengdu | 1 | 24.04 | 24.53 | 23.73 | 24.05 | 25.15 | 24.59 | 25.22 | 25.22 |
|  |  | 2 | 23.83 | 24.05 | 24.10 | 23.77 | 25.03 | 25.61 | 25.23 | 24.89 |
|  |  | 3 | 23.56 | 23.91 | 24.14 | 23.53 | 25.32 | 25.04 | 25.44 | 25.10 |
|  | Kunming | 1 | 32.78 | 32.13 | 32.18 | 32.46 | 33.64 | 33.39 | 33.70 | 33.42 |
|  |  | 2 | 33.19 | 33.29 | 32.74 | 33.24 | 34.09 | 34.32 | 34.24 | 34.35 |
|  |  | 3 | 33.99 | 33.90 | 33.35 | 33.67 | 34.42 | 34.85 | 34.81 | 34.52 |





Figure S1. Quantitative relationships between REs of LMG 9673 or RsPC and soil physicochemical characteristics. Dotted lines indicate 95% confidence bands. Regression analysis was conducted using GraphPad Prism 9.00 (p<0.05). The correlation coefficients and their significance levels are listed in figures.
